# High-Optical-Performance Composite Films of Deep Eutectic Solvent Pretreated-Cellulose Nanofibrils and Fibrous Clay Minerals

**DOI:** 10.1021/acs.biomac.5c00376

**Published:** 2025-09-02

**Authors:** Ricardo O. Almeida, Eduardo Ferraz, Ana Ramos, Verner Håkonsen, Maria L. Puertas, José A. F. Gamelas

**Affiliations:** † Department of Chemical Engineering, CERES, University of Coimbra, Rua Sílvio Lima, Pólo II, 3030-790 Coimbra, Portugal; ‡ TECHN&ART, 70924 Polytechnic Institute of Tomar, Quinta do Contador, Estrada da Serra, 2300-313 Tomar, Portugal; § Department of Chemistry, FibEnTech, University of Beira Interior, Rua Marquês d’Ávila e Bolama, 6201-001 Covilhã, Portugal; ∥ NTNU NanoLab, 8018 Norwegian University of Science and Technology (NTNU), 7491 Trondheim, Norway; ⊥ Research & Technology for New Businesses, TOLSA, SA, Ctra. de Madrid a Rivas Jarama 35, 28031 Madrid, Spain

## Abstract

Cellulose nanofibrils (CNFs) produced via deep eutectic solvent (DES) pretreatment were used, for the first time, to prepare composite films/nanopapers with fibrous clays (sepiolite and palygorskite). Highly transparent films containing up to 50% clay were successfully obtained, avoiding energy-intensive homogenization processes and clay chemical modifications, with absolute transparency losses relative to the transparency of the neat CNF film of ∼15% for 50% sepiolite. Higher transparency losses were found for TEMPO-oxidized CNF and cationic CNF composite films prepared for comparison purposes. Moreover, films with wet micronized sepiolite exhibited higher transparency than those with dry micronized sepiolite or with palygorskite. The combination of very thin, highly functionalized, and dispersible nanofibrils of DES CNF with sepiolite fibers enabled the production of films with a very dense and compact structure, contributing to their high optical performance. The straightforward preparation method and the incorporation of clay minerals allow the reduction of the film production costs.

## Graphical abstract

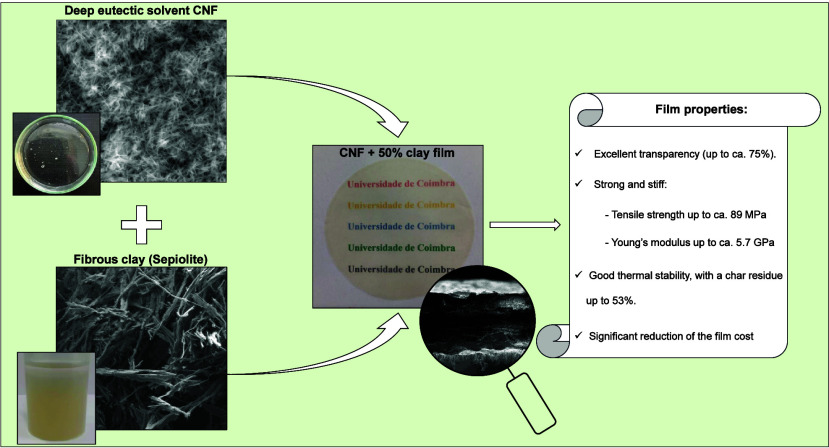

## Introduction

Studies centered on hybrids or composites of cellulose nanofibrils (CNFs) with fiber clay minerals (sepiolite and palygorskite) are nowadays on the research agenda due to the inherent sustainable factors (bio-based and renewable sources) and promising industrial production associated with these materials. In the early studies in the past decade, planar clay minerals have been preferentially explored.^1–5^ However, more recently, fibrous minerals, particularly sepiolite, have also gained attention for the production of composite materials with CNFs due to their high density of silanol groups on the surface and fibrous morphology, which together could favor their interaction with cellulose fibers.^6^ The incorporation of fibrous clays into CNF matrices can impart barrier properties to the composite materials (e.g., oxygen barrier) and increased thermal stability and allow a reduction of the production costs of the final material.^7–9^ Several studies have already reported the production of films/nanopapers^7,8,10–12^ and foams/aerogels^13,14^ based on sepiolite and CNFs.

High transparency is claimed to be one of the most important and distinctive properties of CNF films, enabling their use in several advanced applications. For instance, highly transparent CNF films can be used in electronic devices (e.g., transparent conductive films,^15^ organic light-emitting diodes^16^), energy conversion and storage devices (e.g., solar cells^17^), packaging materials,^18^ and restoration of old paper documents.^19^ Typically, the production of transparent CNF films requires the introduction of charged groups into the surface of cellulose fibers, followed by mechanical homogenization, to obtain individualized CNFs able to form dense films. The shorter the fibrils are and the denser the film is, the less light is scattered and the more transparent is the film. This is the case, for instance, with TEMPO-oxidized CNF,^20^ phosphorylated CNF,^11^ and CNF pretreated with functionalizing deep eutectic solvent (DES).^21^ For instance, in 2009 Fukuzumi et al.^20^ reported the preparation of transparent films using TEMPO-oxidized CNFs with 1.5 mmol/g carboxyl content produced from softwood and hardwood cellulose sources. The transmittance (at 600 nm) was about 90% for the film prepared from softwood cellulose and about 78% for the film prepared from hardwood cellulose. A similar level of transparency (total transmittance near 90%) was reported in 2024 by Almeida et al.^22^ for a film prepared from a CNF produced by pretreating bleached *Eucalyptus* kraft pulp with a DES composed of sulfamic acid and urea.

Although the incorporation of minerals into CNF matrices can improve some properties of CNF films and reduce film preparation costs, it also reduces transparency due to light scattering and absorption phenomena caused by the presence of the mineral particles. To limit this reduction in the film’s transparency, efforts have been undertaken to optimize the mineral dispersion as well as the mineral–CNF composite dispersion before the film formation. Employing high-power homogenization tools such as ultrasonication, high-pressure homogenization, or Ultra-Turrax, and using chemically modified clays, it was possible to mitigate this phenomenon, at least for a low clay content, thus keeping transparency of the CNF-based films reasonably high (>70% in transmittance at 600 nm for 20–25% clay) (e.g., Aulin et al.,^2^ Wu et al.,^3,4^ Ming et al.,^23^ and Liu et al.^24^). To achieve this, highly exfoliated particles are usually required when using planar clays. As few examples, the transmittance at 550 nm (the wavelength corresponding to the maximum sensitivity of the human eye to visible light) was around 85% for a film of carboxymethylated CNF and reduced to ca. 70% after 20% clay (chemically exfoliated vermiculite) incorporation.^2^ In another study, composite films of amino-clay and TEMPO-oxidized CNF showed remarkably high values of transparency, even at high clay contents in the composite, reaching ca. 85% transmittance at 550 nm for 50% clay content (ca. 95% for neat CNF).^24^ However, in this study the authors used a synthetic amino-clay to prepare their composite films and ultrasonication for the clay dispersion, which may explain the observed high transparency values achieved. Recently, Martín-Sampedro et al.^7^ reported hybrid films of CNF and sepiolite with clay content up to 20%, where the total transmittance and haze were measured. It was found that the composite films exhibited significant haze, particularly those with higher amounts of sepiolite. The total transmittance (at 550 nm) of the composites with 20% clay was in the range of 73–80% depending on the CNF type (lignocellulose nanofibrils, cellulose nanofibrils, and TEMPO-oxidized cellulose nanofibrils were used).

In the present work, composite films with high transparency of CNF and clay minerals were prepared, avoiding energy-intensive and difficult to upscale dispersion methods and/or the use of chemically modified clays. To achieve this, two fibrous clay minerals (sepiolite and palygorskite) and a highly fibrillated CNF produced using a DES pretreatment were used to prepare the composite films/nanopapers. For comparison purposes, composite films were also prepared by using TEMPO-oxidized and cationic CNFs. The prepared films were fully characterized for their structural, optical, mechanical, morphological, and thermal properties. Additionally, an assessment of their preparation costs was also conducted. To our knowledge, this work presents the first studies reported so far on composite materials of DES-pretreated nanocelluloses with clay minerals in which remarkable transparency values were obtained, even at high mineral load.

## Materials and Methods

### Clay Minerals

Two sepiolite samples, denoted as SEP A and SEP B, with origin on the deposit of Vallecas-Vicálvaro (Madrid, Spain) and a palygorskite sample, denoted as PAL, collected on the deposit of M’bour, region of Thiès (Senegal), were supplied by Tolsa, SA (Madrid, Spain). SEP A was processed by dry micronization in a jet mill to break the fiber bundles down into micrometer-sized particles. SEP B was wet-micronized to obtain an extensive deagglomeration of the fiber bundles without affecting their aspect ratio. PAL was treated using a roller mill to achieve micrometer-sized particles. Sepiolite and palygorskite were selected for this study due to their morphological similarity to CNFs (thickness of 10–40 nm and length of 1–10 μm) and their high density of surface silanol groups, enabling good compatibility with CNFs.^6,8,9^ Additionally, two sepiolite samples differing in the preprocessing applied by the supplier to the raw material collected on the deposit were used. These samples were expected to show different ability for the production of composite films with CNFs due to differences in their dispersibility in water (with SEP B being easier to disperse).^25^ Field-emission scanning electron microscopy (FE-SEM) images of the three clay mineral samples are presented in Figure 1. The mineralogical, chemical, and physical characterization of these mineral samples can be found elsewhere.^25,26^ This detailed characterization involved X-ray diffraction, X-ray fluorescence, thermal analysis by simultaneous thermogravimetry and differential scanning calorimetry, Fourier transform infrared–attenuated total reflection spectroscopy, laser diffraction particle size analysis, and zeta potential measurements in a wide range of pH.

**Figure 1:**
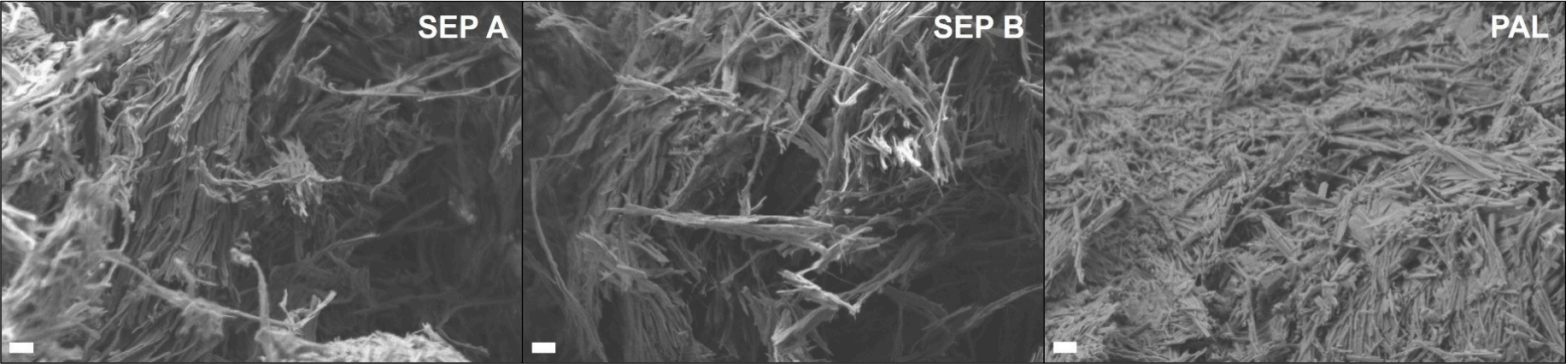
FE-SEM micrographs of the clay mineral samples at 20000× magnification. The scale bars correspond to 200 nm.

### CNF Samples

A highly sulfated CNF was produced, along with a TEMPO-oxidized CNF with high carboxyl content and a cationized CNF for comparison purposes. To produce these different types of CNFs, a bleached *Eucalyptus globulus* kraft pulp (BEKP) with a consistency of 34.5% was used as the starting material. The BEKP was kindly supplied by The Navigator Company and was composed of 73.0 wt % cellulose, 18.3 wt % xylan, and 0.8 wt % soluble lignin (with an undetectable amount of klason lignin).

For the preparation of the sulfated CNF (hereafter denoted as DES CNF), the BEKP was first washed with ethanol, filtered, and dried in an oven at 60 °C for at least 24 h. Subsequently, 10 g of the dried BEKP was treated with a DES mixture of sulfamic acid and urea (1:2 molar ratio), which was previously prepared by magnetic stirring at 80 °C for about 45 min (until a clear solution was obtained). When the dried BEKP was added to the DES (with a concentration of 6.9 wt %), the reaction temperature was increased to 150 °C and maintained for 30 min.^27^ The pretreated fibers were filtered and washed several times with distilled water until the pH of the filtrate was near neutral.

For the TEMPO-oxidized CNF (denoted as TEMPO CNF), a procedure was used that consisted of pretreatment of the BEKP with sodium hypochlorite under alkaline conditions using 2,2,6,6-tetramethylpiperidin-1-oxyl (TEMPO) and sodium bromide as mediators.^28,29^ BEKP (30 g on a dry basis) was treated with 0.48 g of TEMPO, 3 g of NaBr, and 165 mL of NaClO (∼13 mmol of NaClO/g of dry pulp). For the cationic CNF (denoted as Cat CNF), the BEKP was suspended in water (5% consistency) with added NaOH (NaOH/anhydroglucose molar ratio of 2). The suspension was left to stir for 20 min at 20 °C. Then (3-chloro-2-hydroxypropyl)­trimethylammonium chloride (CHPTAC) at a CHPTAC/anhydroglucose molar ratio of 0.5 was added, and the cationization was allowed to proceed for 4 h at 70 °C. Following TEMPO and cationic pretreatments, the pretreated fibers were filtered and washed several times with distilled water until the conductivity of the filtrate was nearly constant.

After the washing step, the pretreated fibers were diluted in water (ca. 1% consistency) and passed twice through a high-pressure homogenizer (GEA Niro Soavi PandaPlus 2000), with the first pass at 500 bar and the second pass at 1000 bar.

A schematic representation of the functional groups introduced into the cellulose structure by the aforementioned chemical pretreatments is shown in Figure 2.

**Figure 2:**
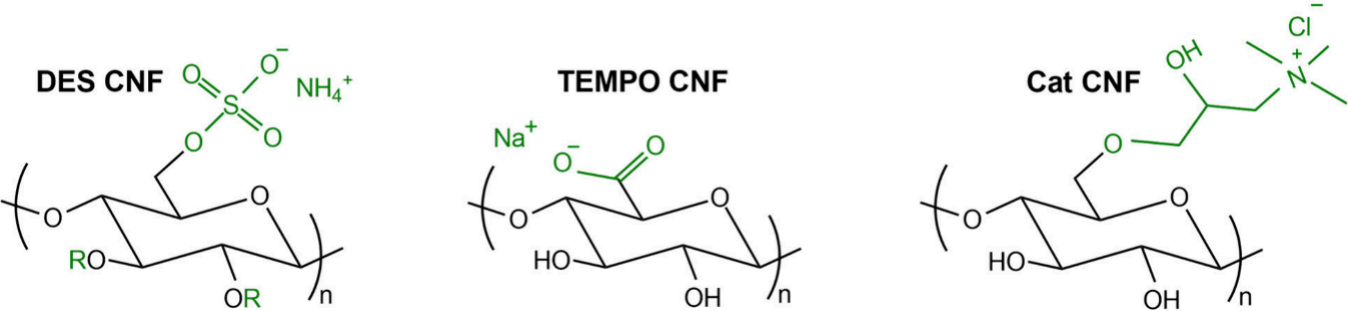
Schematic representation of the surface functional groups in each CNF type.

The resulting DES CNF, TEMPO CNF, and Cat CNF were characterized for their consistency, degree of fibrillation, degree of polymerization of cellulose (cupriethylenediamine method), zeta potential, sulfate group content, carboxyl content, cationic group content, and morphology using previously established protocols (see Supporting Information).^22,30^ The chemicals used for the reactions were supplied by Sigma-Aldrich (Merck), except for sodium hypochlorite (14% active chlorine), which was purchased from VWR Chemicals. Additionally, sulfamic acid (≥99.5%) and urea (≥98.5%, pearls) were both obtained from PanReac AppliChem. Before the TEMPO oxidation and cationization pretreatments, the cellulose fibers were first refined at 4000 rev. in a laboratory PFI beater to make the fibrils more accessible. The properties of the obtained CNFs are summarized in Table 1, and the atomic force microscopy (AFM) images are shown in Figure 3. The AFM images were obtained as described previously.^22^

**Table 1:** Properties of the Produced CNFs

CNF	consistency (%)	degree of fibrillation (%)	degree of polymerization	substituent group content (mmol/g)	Zeta potential (mV)	AFM average diameter (nm)
DES	0.72 ± 0.01	∼100	739 ± 1	2.27 ± 0.04	–67 ± 2	3.5 ± 0.4
TEMPO	0.99 ± 0.03	88.1 ± 2.5	355 ± 6	1.27 ± 0.05	–58 ± 4	5.2 ± 0.3
Cat	0.71 ± 0.02	31.1 ± 0.1	1213 ± 12	0.41 ± 0.01	+40 ± 3	8.7 ± 0.5

**Figure 3:**
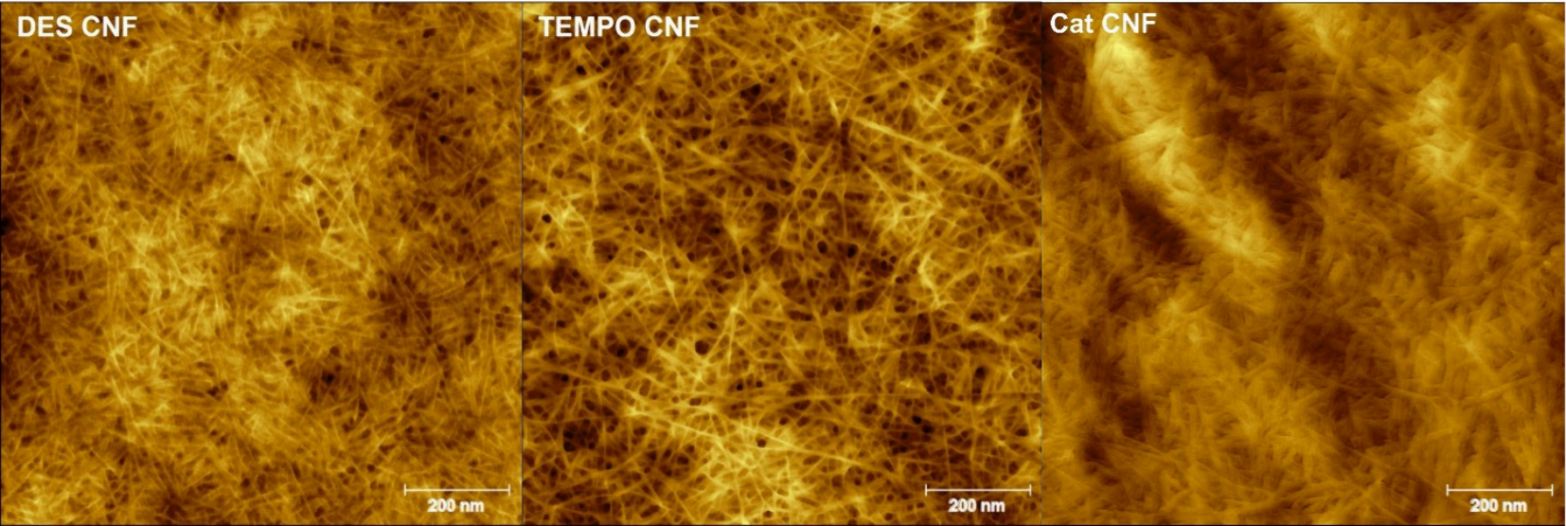
AFM images of DES CNF, TEMPO CNF, and Cat CNF.

### Preparation of the CNF–Clay Composite Films

Initially, aqueous suspensions of the mineral samples at 1% were prepared by stirring them in a Dispermat CV3-PLUS-E high-speed disperser at 5000 rpm for 15 min.

CNF–mineral composite films (nanopapers) with different compositions were prepared by filtration followed by hot pressing.^31^ Films were designed to have a basis weight of ca. 40 g/m^2^ and 10, 20, or 50% mineral content (100% × mineral mass/[mineral mass + CNF mass]). Briefly, each formulation, consisting of a mixture of CNF with mineral sample (SEP A, SEP B, or PAL) was diluted and allowed to disperse for 10 min at 1000 rpm in the Dispermat. The resulting suspension was filtered in a filtration unit from Kimble Ultra-Ware Filtration Systems using PVDF membrane filters (pore size of 0.45 μm and diameter of 90 mm, from Sigma-Aldrich) for the DES CNF-based films and cellulose acetate membrane filters (pore size of 0.45 μm and diameter of 90 mm, from Filtratech) for the TEMPO CNF- and Cat CNF-based films. The PVDF membrane filters were used because it was not possible to detach the DES CNF-based films from cellulose acetate membrane filters. After filtration, the wet cakes retained over the membranes were dried using a Lorentzen & Wettre 257 rapid dryer for laboratory sheets for 10 min at 110 °C, and the resulting films were detached from the membranes. For the DES CNF film series, several drying cycles were performed: the first cycle was performed at 95 °C for 15 min, followed by four cycles at 100 °C for 10 min. In this case, several drying cycles were required since the DES CNF was highly substituted and hydrophilic, making the formed films more challenging to dry compared to the TEMPO and Cat CNF analogues. CNF films without mineral (neat CNF) were also prepared as references.

### Characterization of the CNF–Clay Composite Films

The prepared films were characterized in terms of structural, optical, mechanical, and morphological properties as well as for their thermal behavior (thermogravimetry).

The basis weight of each prepared film was determined by dividing the mass of the dried film by its respective area. The thickness was measured using a Adamel Lhomargy MI 20 micrometer, as the average of five points for each film. Two replicates were averaged.

The tensile properties were determined according to the ISO 1924-1 (1992) standard at 23 ± 1 °C and 50 ± 2% RH using a Thwing-Albert EJA series tensile strength tester. A tensile rate of 5 mm/min and an initial gap between grips of 5 cm were used. Tensile strength, elongation at break, and Young’s modulus were evaluated as the average of four specimens for two replicate films.

The optical properties (transparency and opacity) were accessed using a Technidyne Color Touch 2 spectrophotometer by applying the D65 illuminant (daylight) and 10° observer at a wavelength of 557 nm.

The total transmittance (transparency) of the films was evaluated according to the ISO 22891 (2013) standard, where transparency is defined as follows:

$$\mathrm{transparency}={\left[\left(R_{W}-R_{0}\right)\left(\frac{10000}{R_{\left(W\right)}}-R_{0}\right)\right]}^{1/2}\times 100\%$$

where *R*_W_ (or *R*_∞_) is the reflectance value for the sample backed by a white body, *R*_0_ is the reflectance value for the sample backed by a blackbody, and *R*_(W)_ is the reflectance value of the white body used as backing (90.2%). The average value of two replicate films was taken in all cases.

The opacity of the films was determined according to the ISO 2471 (2008) standard using the following formula:

$$\mathrm{opacity}=\frac{R_{0}}{R_{\infty }}\times 100\%$$

In addition, the UV–vis reflectance spectra were recorded in duplicate on a Jasco V-650 spectrophotometer equipped with an ISV-722 integrating sphere, using barium sulfate as the background. Spectra were obtained in the range of 700 to 250 nm at a scanning speed of 200 nm/min and a bandwidth of 5 nm.

The cross-sectional morphology of the films was evaluated by SEM. The SEM images were captured on a Thermo Fisher Scientific SEM Apreo using an acceleration voltage of 2 kV, a beam current of 13 pA, and a secondary electron detector. The as-prepared films were cut and subjected to cross-sectional imaging without applying any conductive coatings.

Thermal analysis was performed using a TA Instruments SDT Q600 thermal analyzer. The samples were heated under a nitrogen flow (100 mL/min) from room temperature up to 1000 °C at a rate of 10 °C/min. The char residue was estimated on a dry basis of material, that is, by dividing the final weight at 1000 °C by the weight obtained after the loss (at 150 °C) of moisture and adsorbed water.

## Results and Discussion

### Structural Properties of the Composite Films/Nanopapers

Films with an average basis weight of 39 g/m^2^ (±1 g/m^2^) were prepared by filtration followed by hot pressing (Table S1). The thickness of the prepared films ranged from 24 to 70 μm (Table 2), with the films based on DES CNF showing lower thickness values than those based on TEMPO CNF and Cat CNF, averaging 33, 39, and 50 μm, respectively. The lower thickness values observed for the DES CNF-based films resulted from the very high degree of fibrillation and the smallest dimensions of the nanofibrils in this type of CNF, as shown in Table 1 and Figure 3. Additionally, for each type of CNF, the films of CNF with PAL exhibited higher thickness values than those with sepiolite: the thickness of sepiolite-based films was in the range of 26–31 μm for DES CNF, 30–35 μm for TEMPO CNF, and 42–45 μm for Cat CNF, whereas the corresponding films with PAL showed thicknesses of 37–53 μm for DES CNF, 52–62 μm for TEMPO CNF, and 58–70 μm for Cat CNF. It should be noted that the basis weight was kept similar for all the prepared films; therefore, the lower thickness values of the DES CNF films compared to the TEMPO CNF and Cat CNF films reflect their denser and more compact structure. This result was corroborated by the determination of the apparent density of the films, which for the sepiolite composites ranged from 1.3 to 1.5 g/cm^3^ for DES CNF-based films and 1.1 to 1.2 g/cm^3^ for TEMPO CNF-based films and was only 0.9 g/cm^3^ for Cat CNF films (Table 2). Moreover, the use of sepiolite samples also resulted in denser structures compared with the use of PAL (0.6–1.0 g/cm^3^). The lower apparent density values obtained for the PAL-containing films can possibly be attributed to the heterogeneous composition and morphology of the PAL sample,^26^ which did not favor good compatibility with the three CNF types.

**Table 2:** Thickness and Apparent Density of the Different Prepared Films of CNFs with Sepiolite and Palygorskite

			thickness (μm)^a^			apparent density (g/cm^3^)		
film			DES films	TEMPO films	Cat films	DES films	TEMPO films	Cat films
DES	TEMPO	Cat	24.0 ± 1.1	30.7 ± 2.5	48.8 ± 4.3	1.62 ± 0.03	1.23 ± 0.11	0.84 ± 0.07
+10% SEP A	+10% SEP A	+10% SEP A	28.0 ± 0.8	31.8 ± 2.0	44.0 ± 5.0	1.41 ± 0.03	1.20 ± 0.05	0.87 ± 0.04
+20% SEP A	+20% SEP A	+20% SEP A	29.4 ± 0.5	31.3 ± 1.6	44.5 ± 3.2	1.36 ± 001	1.24 ± 0.05	0.87 ± 0.02
+50% SEP A	+50% SEP A	+50% SEP A	31.2 ± 0.4	34.8 ± 1.2	43.4 ± 7.4	1.30 ± 0.00	1.11 ± 0.03	0.90 ± 0.08
+10% SEP B	+10% SEP B	+10% SEP B	26.0 ± 0.8	31.6 ± 1.6	42.2 ± 6.8	1.49 ± 0.08	1.14 ± 0.07	0.94 ± 0.01
+20% SEP B	+20% SEP B	+20% SEP B	26.6 ± 0.8	29.6 ± 0.5	43.6 ± 3.6	1.45 ± 0.05	1.22 ± 0.08	0.93 ± 0.02
+50% SEP B	+50% SEP B	+50% SEP B	29.0 ± 1.6	32.0 ± 0.6	44.8 ± 3.1	1.36 ± 0.02	1.15 ± 0.01	0.90 ± 0.06
+10% PAL	+10% PAL	+10% PAL	36.6 ± 1.2	52.0 ± 1.7	57.5 ± 5.1	1.04 ± 0.00	0.74 ± 0.01	0.69 ± 0.04
+20% PAL	+20% PAL	+20% PAL	46.8 ± 2.1	57.0 ± 1.8	60.6 ± 3.3	0.80 ± 0.06	0.67 ± 0.00	0.66 ± 0.01
+50% PAL	+50% PAL	+50% PAL	53.2 ± 1.4	61.6 ± 1.7	70.1 ± 4.1	0.71 ± 0.00	0.62 ± 0.02	0.56 ± 0.06

a Measurements were made at 23 °C and 50% RH.

### Optical Properties

The transparency of the films was evaluated, and the results for all films are presented in Figure 4. The digital photographs of the series of the most transparent films are shown in Figure 5. The transparency of the films with solely CNF (DES, TEMPO, and Cat) was high, particularly that of the films of DES CNF and TEMPO CNF, which exhibited nearly the same transparency value (88.3%). The larger transparency achieved with the films of DES CNF and TEMPO CNF resulted from the higher fibrillation (related to the high contents in sulfate and carboxyl groups, respectively) and the presence of shorter fibers (associated with the lower degree of polymerization) in DES and TEMPO CNFs compared to Cat CNF. The presence of cellulose nanofibers with lower size than the wavelength of visible light (400–700 nm) allows for the high transmission of the visible radiation through the film. As expected, the incorporation of minerals in the CNF-based films promotes a decrease in transparency: the presence of opaque and colored mineral particles reduces the light transmission due to scattering and absorption, thus providing lower values of film transparency (total transmittance). However, the loss in transparency was found to be dependent on several factors, as follows. The main factor was found to be the clay content of the composite film. As the clay content is increased, the transparency decreases (Figure 4). In general, the loss in transparency was not high for a clay content of 10%, with maximum absolute losses of ca. 10%. For 20% clay content, transparency could drop up to 20% in absolute loss in comparison to the CNF-only film. For 50% clay content, the final transparency values were generally low, and losses in transparency higher than 30% were found. However, for the films of DES CNF with 50% clay content, the transparency losses (versus transparency of neat CNF) were significantly lower. In this sense, the type of CNF also had a significant influence in the transparency of the composite films. The composite films with DES CNF consistently exhibited lower transparency decays compared to the analogous TEMPO CNF and Cat CNF films, regardless of the type and content of the three clay mineral samples used. In fact, for lower clay contents (10 and 20%), the transparency losses were in the following increasing order: DES CNF-based films < TEMPO CNF-based films ≤ Cat CNF-based films. For 50% SEP B, a similar trend was observed, but with 50% SEP A and PAL, the order shifted slightly to DES CNF-based films < Cat CNF-based films < TEMPO CNF-based films. The third factor limiting the film transparency was the mineral sample used in the composite film’s preparation. Interesting results were obtained on this matter. For films prepared with the more fibrillated CNF (DES CNF), the incorporation of either SEP A or SEP B caused only minor decrements in transparency (transparencies of 88.3% for the DES CNF-only film, 86.9% for 10% SEP B, 85.8% for 10% SEP A, 85.7% for 20% SEP B, 83.4% for 20% SEP A, 73.2% for 50% SEP B, and 72.2% for 50% SEP A), which were less pronounced than those observed with PAL (Figure 4). On the other hand, for TEMPO CNF-based films, larger differences were found between the incorporation of SEP A and SEP B, regardless of the clay content, with lower decrements in transparency noted when using SEP B (transparencies of 88.3% for the TEMPO CNF-only film and 85.1% for 10% SEP B versus 80.6% for 10% SEP A, 79.0% for 20% SEP B versus 71.2% for 20% SEP A, and 48.8% for 50% SEP B versus 37.6% for 50% SEP A). However, composite films of Cat CNF with SEP A and SEP B, at the same mineral content, showed reasonably similar transparency values (respectively, 73.4% and 71.6% for 10% mineral and 65.7% and 66.4% for 20% mineral), falling within the same range of the values obtained for the films with PAL (Figure 4). This suggests that in the case of Cat CNF-based films the three fibrous mineral samples behaved similarly regarding transparency.

**Figure 4:**
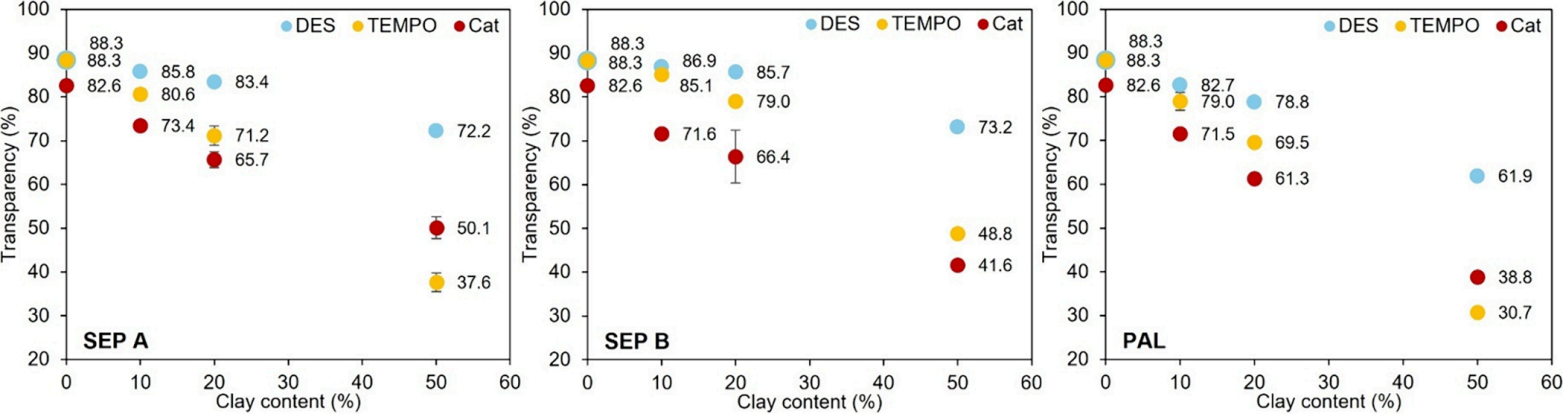
Transparency (total transmittance at 557 nm) of the obtained CNF–clay composite films.

**Figure 5:**
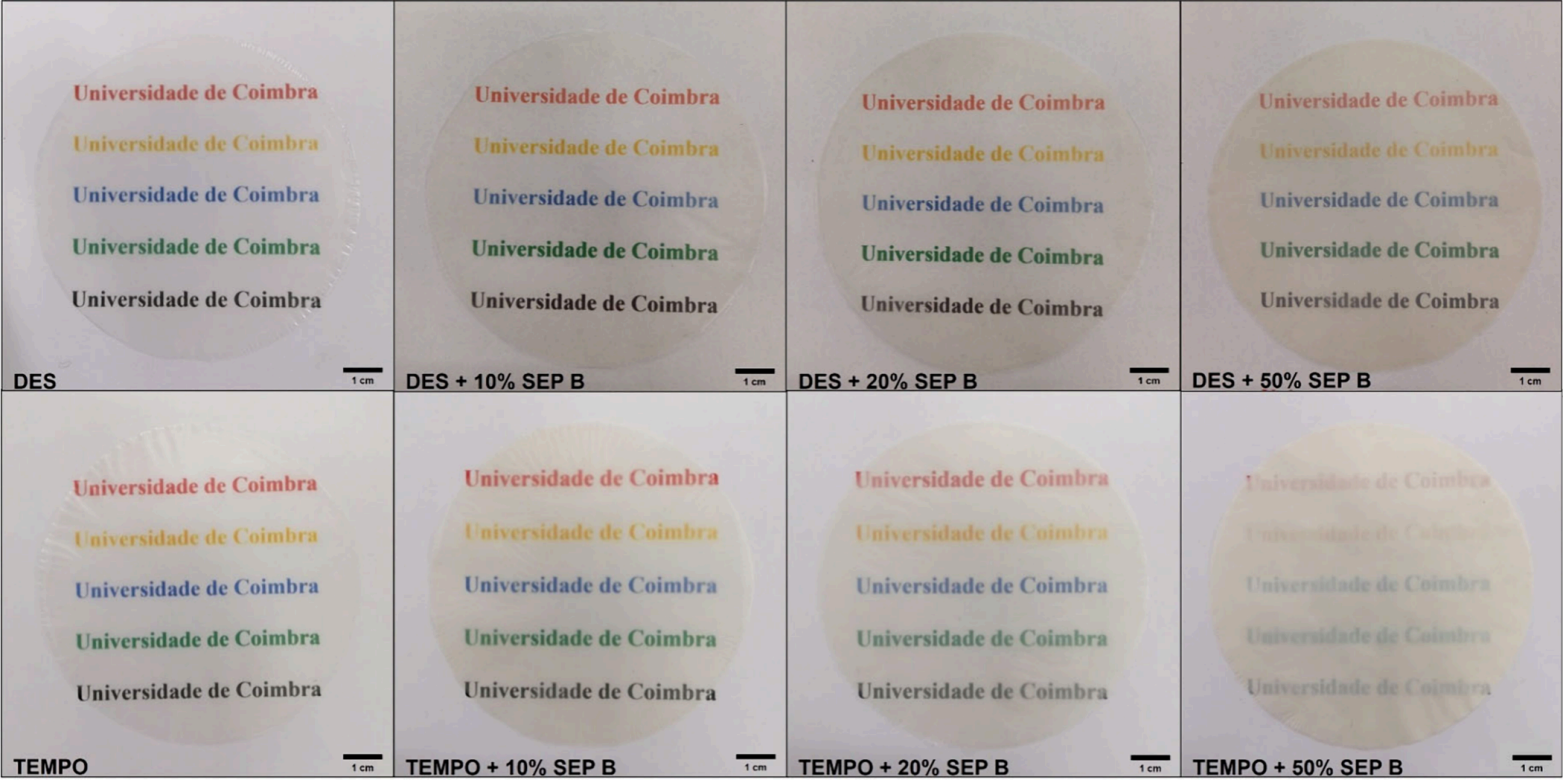
Digital photos of the series of the most transparent CNF–clay composite films.

The composite films prepared with DES CNF and SEP B were those showing higher transparency values. When DES CNF or TEMPO CNF is used, both the CNF and mineral particles are negatively charged, enabling good dispersion of the two composite components upon mixing. However, the fibrils of the DES CNF are slightly thinner (average diameter of 3.5 nm) and shorter (as can be seen in Figure 3) than those of the TEMPO CNF (average diameter of 5.2 nm), which enhanced the compatibility with the mineral particles, thus affording more compact structures and more transparent films. Denser films were indeed obtained using DES CNF combined with SEP B (1.4–1.5 g/cm^3^) compared with those using TEMPO CNF (1.1–1.2 g/cm^3^) and Cat CNF (0.9 g/cm^3^). The DES CNF also has the highest degree of fibrillation, near 100%, meaning that almost all particles in this CNF sample are nanosized. Although this was not reflected in the transparency of the neat CNFs (as measured by the total transmittance values), it was positively reflected in the transparency of the CNF–mineral composite films. Accordingly, remarkable transparencies were obtained for the DES CNF-based films even at a high clay content of 50%. In addition, of the three mineral samples evaluated, SEP B is the easiest to disperse in water, and as reported previously, reasonably stable suspensions under near neutral pH can be obtained using only high-speed homogenization, which is not possible to achieve with SEP A and PAL (Figure 6) that require higher-energy treatments. This is related to the fact that SEP B presents more disaggregated fiber bundles than SEP A and PAL and more negative zeta potential in water (ca. −20 mV for SEP B versus ca. −12 mV for SEP A and −17 mV for PAL)^25,26^ .Concomitantly, SEP B may also improve the dispersion of the CNF particles, even considering that DES and TEMPO CNFs are already well-dispersed in water due to their high negative charge and small fibril size. This positive effect was actually confirmed for the composite films of TEMPO CNF with SEP B compared with those with SEP A, as more transparent films were obtained using SEP B for a given mineral content. With Cat CNF, differences between SEP A and SEP B almost vanished. Here, the electric charges of the mineral and CNF surfaces have opposite signs. When the mineral and CNF particles are held together in the composite, it can be assumed that the positive charges on the surface of the cationic CNF enable good cohesion in the composite film by favoring interaction with the negatively charged particles of the mineral. In this case, the dominant effect seems to be the CNF, and the differences in size and dispersion ability of the mineral particles (always negatively charged) have a minor influence on the composite transparency, except when the clay content is increased to 50%. Of all the results obtained, the composite film formed by DES CNF and 10% SEP B exhibited the lowest transparency decrease compared to the neat DES CNF film, attaining an outstanding transparency value of 87%. The films of DES CNF + 20% SEP B, DES CNF + 10% SEP A, and DES CNF + 20% SEP A also achieved transparency values very close to that of the neat DES CNF film, representing minimal transparency losses of less than 5%. However, taking into account the mineral load, the most remarkable results were achieved with DES CNF + 50% sepiolite, whose films showed transparencies higher than 72% with both SEP A and SEP B. Additionally, it should also be highlighted that the film composed of TEMPO CNF + 10% SEP B reached a transparency of 85% (representing a diminishment in transparency of only 3% compared to the TEMPO CNF-only film) and the film of TEMPO CNF + 20% SEP B still showed a remarkable value of transparency (79%).

**Figure 6:**
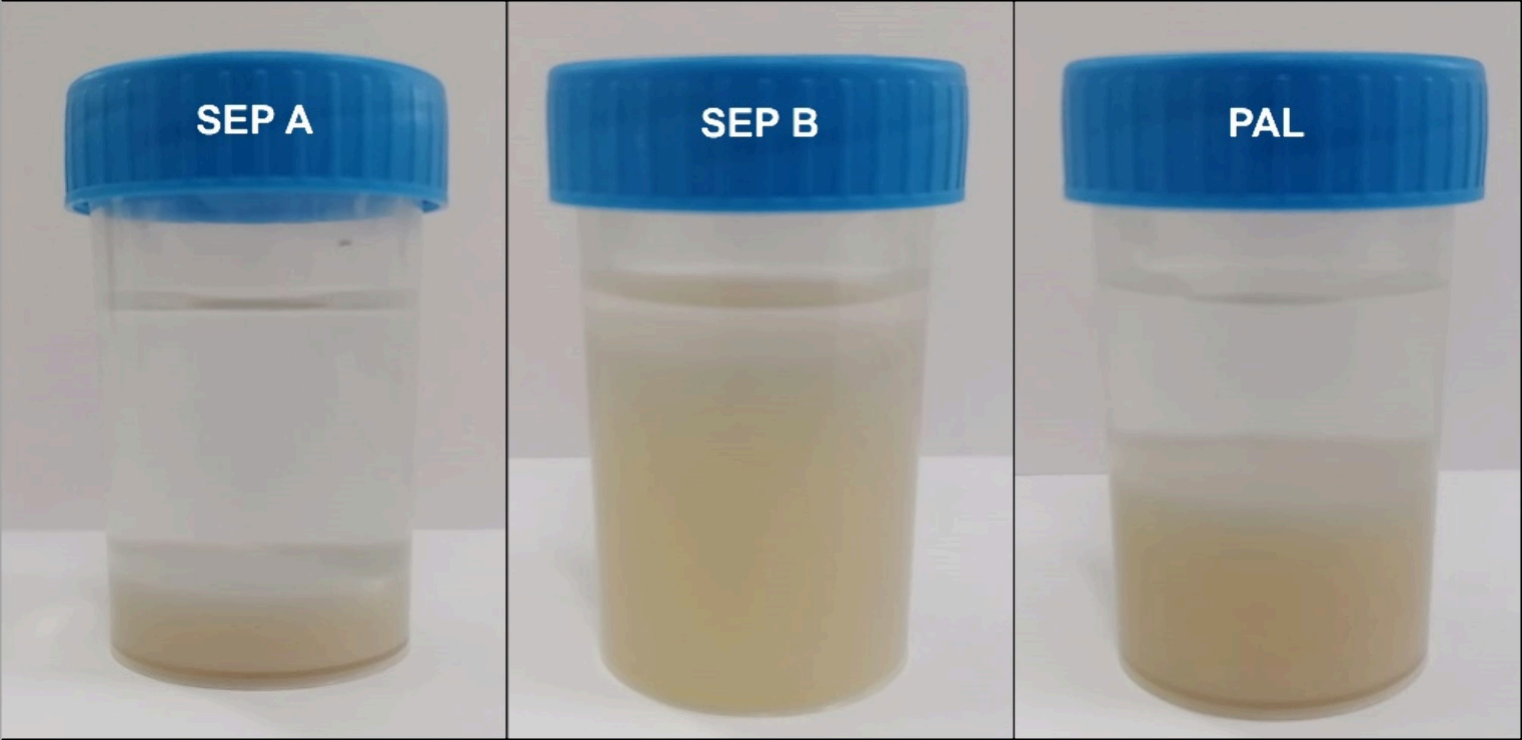
Photographs of the three mineral dispersions after 1 week of preparation.

The opacity of the films was also measured, corroborating the transparency data and confirming the expected inverse relationship between the transparency and opacity (Figure S1). According to the above-mentioned trend for transparency, the films of DES CNF were the least opaque, followed by the TEMPO CNF- and Cat CNF-based films, for a given mineral sample and mineral content. The reflectance spectra recorded for the CNF composite films with SEP B (Figure 7) are in good agreement with the transparency values obtained. Across the entire visible region, the DES CNF-based films exhibited lower reflectance values compared with TEMPO CNF- and Cat CNF-based films. Additionally, irrespective of the CNF type, increasing the SEP B content resulted in higher reflectance values for the composite films; however, this increase was less pronounced in the DES CNF series. Similar trends were also observed in the reflectance spectra of the CNF composite films with SEP A and PAL (Figure S2).

**Figure 7:**
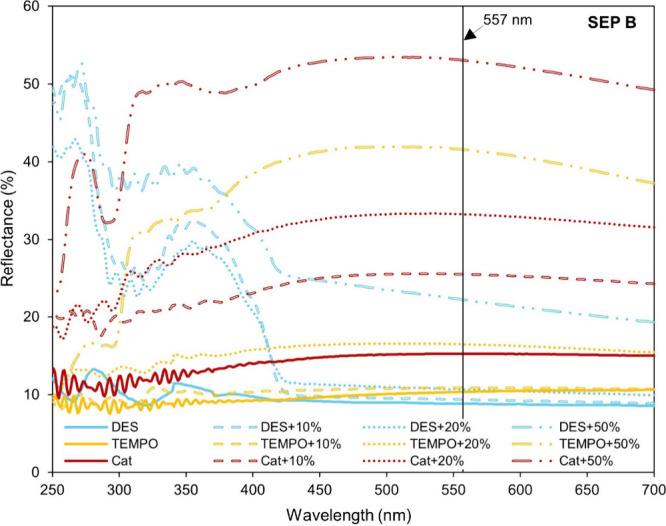
UV–vis reflectance spectra of CNF-SEP B composite films.

When clays are incorporated into the CNF matrix, typically the transparency should decrease, and this is the most common behavior observed with both planar and fibrous clays (e.g., Aulin et al.^2^ and Wu et al.^3^). Several strategies have been used to overcome this problem. As mentioned in the Introduction, these include the use of chemically modified clays or planar clay samples with very small platelet sizes and/or applying successive steps of homogenization/dispersion of the clay particles and of the clay–CNF mixtures (e.g., Wu et al.,^4^ Ming et al.,^23^ and Liu et al.^24^). Although they proved to be effective in improving transparency, mainly by reducing the particle size and enabling better particle (individual and composite) dispersion, some of those strategies are not easy to implement and upscale, as is the case of ultrasonication, and may involve considerable amounts of energy, which is a limitation for their real application. In the present work, the use of intensive homogenization steps was avoided. Using DES CNF combined with sepiolite dispersed in water using high-speed shearing was found to be a good approach for the purpose of achieving high transparency values in the final composite films/nanopapers. The combination of an excellent dispersion and very small particle size of the DES CNF with a good dispersion of the sepiolite particles (better in the case of SEP B) guaranteed high values of transparency in the composite films up to 50% clay content. Not as good results were obtained for the TEMPO CNF-based films due to the lower degree of fibrillation (larger particle size) of the TEMPO CNF; however, the easier to disperse SEP B (wet-micronized) improved to a large extent the transparency of the films compared to SEP A (dry-micronized). The present results for the TEMPO CNF-based films are similar to those obtained by Martín-Sampedro et al.,^7^ who used a different strategy to prepare CNF–sepiolite composites. With a TEMPO-oxidized CNF, the authors were able to produce films (by solvent casting) with a basis weight of ca. 10 g/m^2^ and a thickness of ca. 7 μm, showing a transparency of 80% for 20% clay and 87% for 10% clay, whereas the neat CNF showed a transparency of 90%. The present TEMPO CNF-based films, in spite of having higher basis weight and being thicker (≥30 μm), showed similar transparency values for the same clay content. For DES CNF–clay composites, here presented for the first time, the transmittances at 10–50% clay were higher than those reported by Aulin et al.^2^ and Wu et al.^3^ when using planar clays and other CNF types. Others have reported transmittances higher than 80% in the 550–600 nm range for composites of planar clays as well and TEMPO CNF at 50% clay content,^4,23,24^ while in the present work ca. 73% was obtained for a similar clay content. However, in all of these previous reports, cumbersome steps of dispersion/homogenization were applied. Even so, the transparency loss of ca. 15% (versus neat CNF) observed here for the composites of DES CNF with 50% sepiolite is not far from the transparency loss of 10% (from 95 to 85%) previously reported by Liu et al.,^24^ who prepared composites of amino-clay and TEMPO CNF under more sophisticated conditions of mineral and CNF–mineral dispersion. To prepare those composite films, first a synthetic amino-clay was obtained by reaction of magnesium chloride with (3-aminopropyl)­triethoxysilane, and then the amino-clay was exfoliated by ultrasonication and mixed with TEMPO CNF using an Ultra-Turrax homogenizer. Only after these multiple steps were the films prepared by vacuum filtration. In the present work, we were able to obtain highly transparent composite films simply by using natural clay minerals in their supplied form and a common high-speed disperser for mixing the components. In this context, our films have great potential for large-scale production. Because of their high transparency, the CNF–clay composites are promising candidates for, e.g., emerging applications in flexible and transparent electronics^16,17^ and restoration/conservation of old paper documents as potential substitutes for Japanese papers.^22^ Table 3 summarizes a comparison between the most transparent CNF–clay composites prepared in this study and those reported in the literature.

**Table 3:** Comparison of Optical Transmittance of CNF–Clay Composite Films

CNF type	clay mineral		dispersion method	transparency (%)		ref
sulfated (DES)	10%	sepiolite	high-speed disperser (Dispermat)	86.9	(557 nm)	this work
	20%			85.7		
	50%			73.2		
phosphorylated	10%	sepiolite	ultrasonication	∼75^a^	(550 nm)	^11^
			Ultra-Turrax			
TEMPO	10%	sepiolite	ultrasonication	86.6	(550 nm)	^7^
	20%		Ultra-Turrax	80.0		
TEMPO	50%	synthetic amino-clay	ultrasonication	∼85^a^	(550 nm)	^24^
			Ultra-Turrax			
TEMPO	10%	montmorillonite	ultrasonication	∼78^a^	(550 nm)	^3^
	20%			∼40^a^		
carboxymethylated	10%	vermiculite	high-pressure homogenization	∼70^a^	(550 nm)	^2^
	20%			∼70^a^		
TEMPO	10%	saponite	homogenization	∼83^a^	(550 nm)	^4^
	50%		ultrasonication	∼83^a^		

a Value extracted from graphical data.

### Mechanical Properties

The tensile properties of the films were also evaluated, and the main results for the tensile strength, elongation at break, and elastic (or Young’s) modulus are presented in Figures 8 and S3. The films of only CNF showed comparable tensile strength values for TEMPO CNF and Cat CNF (71 and 83 MPa, respectively), whereas the tensile strength of the pristine DES CNF film was slightly higher (114 MPa). The higher tensile strength of the DES CNF film can be attributed to the higher degree of fibrillation of DES CNF, which enabled the formation of a stronger hydrogen-bonding network within the film. On the other hand, the neat TEMPO CNF film exhibited a lower tensile strength compared to values typically reported for similar films in the literature.^20,32,33^ Multiple factors, often interdependent, can influence the tensile properties of CNF films, making direct comparisons among different studies difficult. Nevertheless, we believe that the lower tensile strength obtained for our TEMPO CNF film may be attributed to the drying conditions used during film preparation. The drying step is also of major importance to the mechanical properties of CNF films and should be optimized in the future.

**Figure 8:**
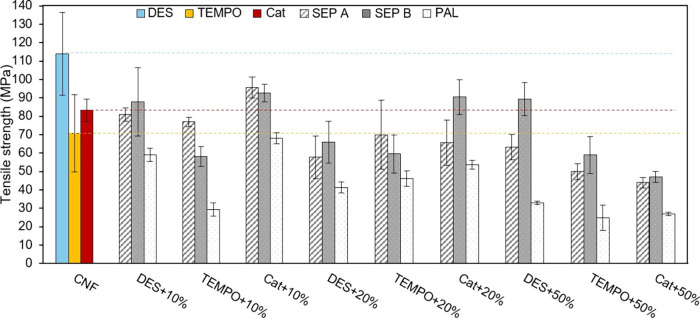
Tensile strength of the CNF–clay composite films (measurements at 23 °C and 50% RH).

After mineral incorporation up to 20% clay content, there was not a systematic decrease in the tensile strength of the TEMPO CNF- and Cat CNF-based films. In fact, even some slight improvements could be observed. For TEMPO CNF and SEP A films at 10 and 20% clay, the tensile strength (77 and 70 MPa, respectively) was roughly the same as that measured for the neat CNF film (taking into account the standard deviations). These tensile strength values are comparable to those reported by Martín-Sampedro et al.,^7^ who achieved tensile strengths of ca. 85.5 and 79 MPa for hybrid nanopapers composed of TEMPO CNF and 10–20% sepiolite. For the analogous films of TEMPO CNF with SEP B, an apparent (but not statistically significant) decrease in the tensile strength to final values of about 60 MPa was observed. Interestingly, for 50% SEP A and 50% SEP B, the tensile strength was in the range of 50–60 MPa, only around 10–20 MPa lower than that of the TEMPO CNF-only film (ca. 70 MPa). For the Cat CNF series, some apparent slight increments in the tensile strength were observed, for instance, with 10% SEP A (from 83 to 96 MPa) and 10% SEP B (to 93 MPa). These increments may result from the electrostatic interactions (attraction) established between the positively charged Cat CNF and the negatively charged sepiolite fibers, which create strong interfacial bonding and enhance stress transfer under tensile loading. On the other hand, for the DES CNF-based films, a decrease in tensile strength was observed after the incorporation of SEP A and SEP B up to 20%: the tensile strength was reduced from 114 MPa to 81–88 MPa for 10% sepiolite and 58–66 MPa for 20% sepiolite. However, the further addition of SEP A and SEP B up to 50% clay content did not compromise the tensile strength of the DES CNF-based films. In fact, for 50% SEP B, the tensile strength was even higher than that obtained for the corresponding film with 20% clay (89 and 66 MPa, respectively), and comparable tensile strengths were demonstrated for the DES CNF-based films with 20 and 50% SEP A. Additionally, the use of PAL in the composite films with DES CNF led to a significant decrease in the tensile strength comparing to the values obtained for the analogous films with SEP A and SEP B. This trend was consistent across the three types of nanocelluloses. Using PAL always provided lower tensile strength values at any specific clay content considered, as shown in Figure 8, indicating the poorer quality of the films obtained using this mineral sample. The PAL sample used in this work was actually a complex mixture of mineral phases, including not only palygorskite as the dominant phase but also quartz, calcite, dolomite, and sepiolite.^26^ It is very likely that this mineralogic composition and morphological variety do not result in good compatibility with the CNFs. This was indeed reflected in the lower apparent density values of the films with PAL (Table 2) and in the lower tensile strength values exhibited.

The elongation at break differed significantly between the CNF films, being mainly dependent on the CNF type. The elongation at break values for the DES CNF films did not exceed 8%, with the pristine DES CNF film exhibiting an elongation of 6.3%. Lower elongation at break values were obtained for the films of TEMPO CNF, with all values below 4%. Interestingly, the DES CNF films with 50% sepiolite showed elongation values similar to that of the DES CNF-only film (7.2% for SEP A and 6.4% for SEP B). For the Cat CNF-based films, the elongation at break reached significantly higher values compared to those of DES CNF and TEMPO CNF films in either the absence or presence of the fibrous minerals. The neat CNF film showed an elongation of 18.5%, and after fibrous clay (SEP A, SEP B, and PAL) incorporation up to 20%, the values were in the range of 14–18%. However, a trend of decreasing elongation with increasing mineral incorporation for the Cat CNF-based films was noted, particularly when the clay content was increased to 50%. The overall results show that the produced films of Cat CNF are more ductile than those of DES CNF and TEMPO CNF. This behavior was previously referred as being characteristic of cationic CNF-based films.^8^ From the FE-SEM images (Figure 9), it was observed that neat DES CNF and TEMPO CNF films exhibited a more ordered cross-sectional structure (stratified) compared to the neat Cat CNF film, which displayed a more irregular structure with a less aligned fibril arrangement. This irregularity could allow some mobility of the fibrils under stress, thereby contributing to the higher ductility observed for the Cat CNF-based films. Composite films of mechanical, enzymatic, and TEMPO-oxidized CNFs have shown lower values of elongation.^7,8,10,34^ The trend of decreasing elongation with mineral incorporation was observed only for the Cat CNF-based films. Since neat TEMPO CNF and DES CNF films, particularly TEMPO CNF, already have low elongation at break values (1.5% and 6.3%, respectively), the incorporation of rigid clay minerals does not result in a significant variation of the elongation at break, contrary to what occurs with Cat CNF, which possesses a higher elongation at break (18.5%).

**Figure 9:**
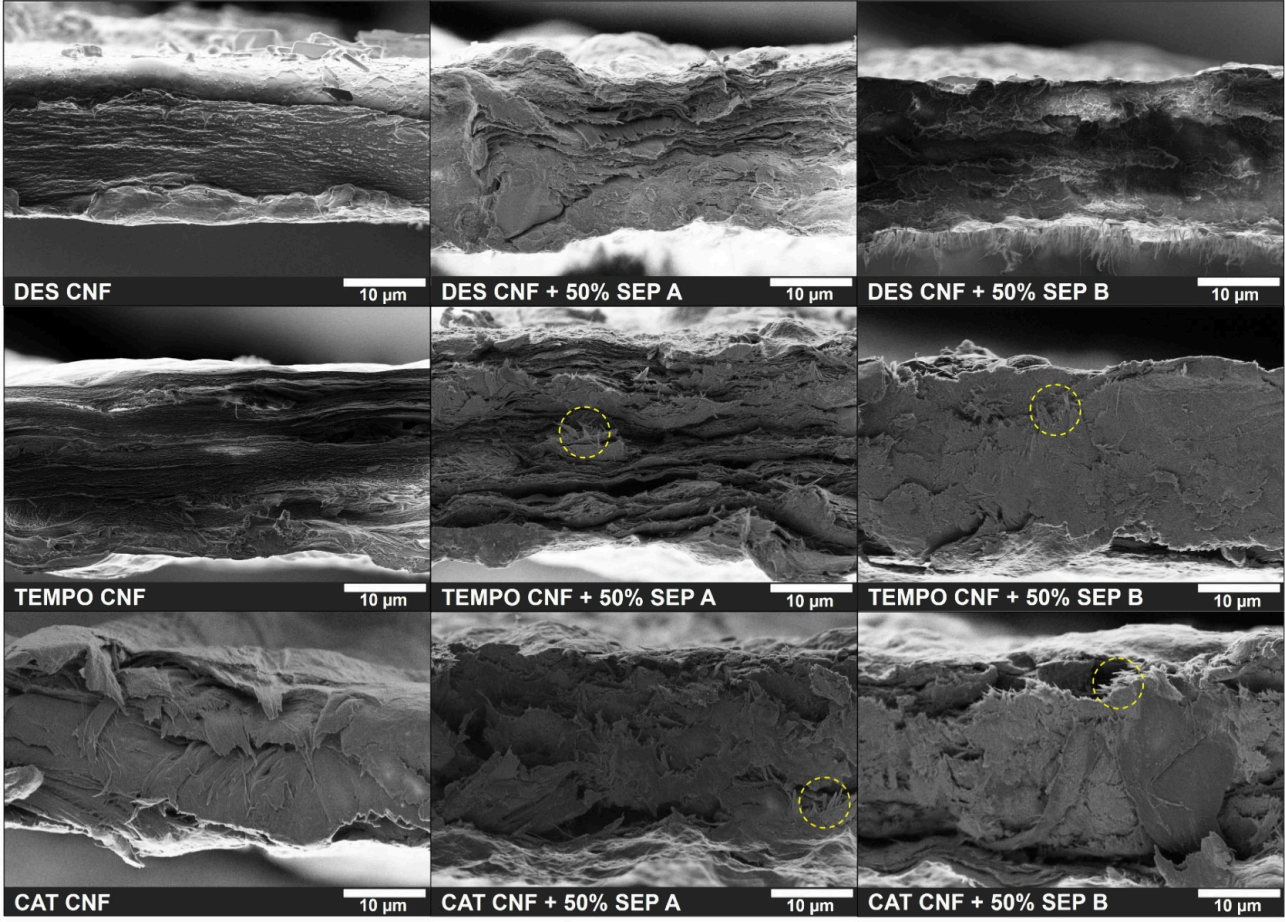
FE-SEM images (magnification of 4000×) of the cross section of the films of DES CNF, TEMPO CNF, and CAT CNF with 50% sepiolite and the corresponding films of neat CNFs.

The Young’s modulus was similar for the DES CNF- and TEMPO CNF-only films (5.4 and 5.7 GPa, respectively) and dropped to less than half (2.4 GPa) for the film of only Cat CNF. These values reflect the significantly higher elongation at break obtained for the latter CNF. With the mineral incorporation, there was a trend of decreasing Young’s modulus in the series of DES CNF films when SEP A and PAL were used, although with SEP B, regardless of the clay content, the Young’s modulus did not change significantly compared to that of the neat DES CNF film. In contrast, the incorporation of SEP A into the TEMPO CNF film series did not affect much the Young’s modulus for any SEP A content, whereas with SEP B and PAL there was a diminishment of the Young’s modulus. The composite films with Cat CNF showed typically lower Young’s modulus than the corresponding films of DES CNF and TEMPO CNF for the same clay sample and clay content. Because of the presence of Cat CNF, the Young’s modulus values of these composite films were limited to maximum values of ∼3.5 GPa. Contrary to the DES CNF and TEMPO CNF films, there was a trend in increasing Young’s modulus with mineral incorporation for the Cat CNF films. Since the neat Cat CNF film exhibited a relatively low Young’s modulus, the incorporation of rigid clay minerals could lead to an increase in this property. This effect was not observed for the DES CNF and TEMPO CNF films because the neat CNF films already exhibited relatively high Young’s modulus values. This observation is consistent with findings in the literature: in studies where an increase in Young’s modulus was reported for CNF–sepiolite composite films, the corresponding neat CNF films had relatively low Young’s modulus values.^10,34^ In contrast, in studies where the neat CNF films already possessed high stiffness, sepiolite addition often resulted in no significant change^11^ or resulted even in a decrease^12^ in Young’s modulus.

In sum, the tensile properties of the composite films with sepiolite samples were not far from those of the neat CNF films, and it was possible to obtain very good film performance even at a clay content of 50%. On the other hand, with the present palygorskite sample used in this work, all the main properties were negatively affected, even at low mineral content, showing the poor ability of this mineral sample to produce CNF composites under the applied conditions of film preparation. The Cat CNF-based films were more ductile and less stiff than the corresponding DES CNF- and TEMPO CNF-based films.

### Morphological Properties

The morphology of the composite films with SEP A and SEP B was evaluated by electron microscopy, and the resulting SEM cross-sectional micrographs are shown in Figure 9. The SEM images of the composite films prepared with PAL are not included due to their overall poorer optical and mechanical properties.

The SEM images of the neat CNF films clearly reveal that the DES CNF film has a more compact and uniform cross section compared with the neat films of TEMPO and Cat CNFs. Moreover, the TEMPO CNF film exhibited a relatively well-organized stratified structure, while the Cat CNF film showed a more irregular structure without a specific pattern. In the cross section of the Cat CNF film, some “bending fibers” were observed, indicating a more malleable film. This structural characteristic could be related to the higher elongation at break value obtained for the Cat CNF compared to the other CNFs, as mentioned above. When 50% sepiolite (SEP A or SEP B) is added, the structural trend of the neat CNF films is maintained; that is, the composite films of DES CNF retain a more closed and compact structure compared with the composite films of TEMPO and Cat CNFs, in agreement with the higher apparent density values determined for the DES-based films. In the TEMPO CNF and Cat CNF composite films, sepiolite rods can be identified in the SEM images (highlighted by yellow dashed circles in Figure 9), which are not observable in the DES CNF films with SEP A or SEP B. The absence of visible sepiolite rods in the DES CNF composite films indicates that very good dispersion was achieved between the two components. Additionally, the cross sections of the composite films of DES and TEMPO CNFs with SEP B are slightly more compact than those of the corresponding films with SEP A, which is in line with the higher transparency and apparent density values obtained when 50% SEP B was used. The very compact structure of DES CNF and SEP B-containing films also suggests films with lower porosity and fewer sites for light scattering, resulting in the high transparency reported above.

### Thermal Stability and Degradation Studies

The films were analyzed for their thermal behavior, including onset temperature of degradation (*T*_on_), the temperature(s) of maximum degradation rate (*T*_max_), and the amount of char residue (%). The results obtained are compiled in Table 4. The thermograms and corresponding derivative curves of illustrative examples are shown in Figure S4. In all cases, degradation of cellulosic chains, unsubstituted or substituted by sulfate groups (DES CNF), carboxyl groups (TEMPO CNF) or alkylammonium groups (Cat CNF) is the limiting step of the film’s degradation. Accordingly, *T*_on_ (Table 4) was lower for the films with DES CNF and TEMPO CNF due to the presence of a high content of sulfate and carboxyl groups, respectively, which start their degradation earlier.^8,35^ The neat DES CNF film started degrading at ca. 189 °C (*T*_on_); however, after the incorporation of 10 and 20% clay, *T*_on_ decreased to 167–170 °C. When the clay content was 50%, the thermal stability of the DES CNF composite films was similar to that of the DES CNF-only film. Similarly, the degradation of the neat TEMPO CNF film (with a high carboxyl content) started at ca. 191 °C; however, in this case, the mineral incorporation slightly increased *T*_on_ (up to ca. 10 °C), with slight variations depending on the clay sample and clay content, showing a positive effect of the mineral presence on the thermal stability of the composite films. In the case of Cat CNF-based films, *T*_on_ was 225 °C for the Cat CNF-only film, and after fibrous clay incorporation, it could be even slightly increased for the films with 10 and 20% clay content.

**Table 4:** Thermogravimetric Data of Films of DES CNF, TEMPO CNF, and Cat CNF with Sepiolite and Palygorskite

film	*T*_on_ (°C)^a^	*T*_max_ (°C)^b^	char residue (%)^c^
DES	189	195, 248	24.7
DES + 10% SEP A	167	170, 195, 245	34.7
DES + 20% SEP A	167	171, 198, 241	42.8
DES + 50% SEP A	190	194, 290	59.0
DES + 10% SEP B	167	171, 198, 236	29.7
DES + 20% SEP B	170	175, 240	41.9
DES + 50% SEP B	189	194, 244	53.0
DES + 10% PAL	170	175, 198, 242	34.0
DES + 20% PAL	168	173, 194, 242	39.6
DES + 50% PAL	183	189, 250	54.3
TEMPO	191	255, 305	21.7
TEMPO + 10% SEP A	201	244, 302	35.8
TEMPO + 20% SEP A	194	249, 307	40.0
TEMPO + 50% SEP A	202	269, 314	55.0
TEMPO + 10% SEP B	188	248, 305	33.8
TEMPO + 20% SEP B	197	243, 307	38.5
TEMPO + 50% SEP B	198	260, 321	52.3
TEMPO + 10% PAL	194	248, 305	29.5
TEMPO + 20% PAL	200	254, 311	38.0
TEMPO + 50% PAL	196	280 (sh), 321	47.1
Cat	225	314, 345	17.6
Cat + 10% SEP A	232	309, 347	26.3
Cat + 20% SEP A	231	311, 347	32.3
Cat + 50% SEP A	223	304, 330	50.3
Cat + 10% SEP B	227	313, 349	21.0
Cat + 20% SEP B	226	312, 348	32.8
Cat + 50% SEP B	214	335	48.0
Cat + 10% PAL	237	305, 341 (sh)	24.9
Cat + 20% PAL	233	302, 343	30.7
Cat + 50% PAL	210	336	46.0

a Temperature corresponding to the onset of material degradation. The estimated error is ca. 3 °C.

b Temperature(s) corresponding to the maximum degradation rate (based on derivative curve). The estimated error is ca. 3 °C.

c Final residue at 1000 °C relative to the mass obtained at 150 °C (dry basis): (*m*_f_/*m*_150_) × 100%. The estimated error is ca. 0.5%.

For the temperature of maximum degradation rate, *T*_max_ (Table 4), two degradation peaks were found at 195 and 248 °C for the DES CNF-only film. At 10 and 20% clay content, these two peaks were also observed for the DES CNF composite films, with small variations in their position. Nevertheless, an additional degradation peak appeared at lower temperatures, close to *T*_on_ for each DES CNF composite film. This result was in line with the *T*_on_ values, showing an apparent decrease in the thermal stability for up to 20% clay incorporation. However, for 50% mineral incorporation, this extra peak was not noted, and only two degradation peaks were found, similar to the behavior of the pristine DES CNF film (Table 4). Two degradation peaks were also observed between 200 and 400 °C in the derivative curves of the TEMPO CNF films. For the TEMPO CNF-only film, these peaks were found at much higher temperatures (255 and 305 °C) than those of the DES CNF-only film (195 and 248 °C). With the mineral incorporation, at 10 and 20% clay content, the former peak (255 °C) was slightly shifted to lower temperature; however, for the highest clay content (50%), this peak was shifted to higher temperature (from 255 up to 280 °C). The peak at *T* > 300 °C did not change much when the clay content was 10–20%, but again, the temperature of this peak increased for 50% content (from 305 °C up to 321 °C). Therefore, for the higher mineral content, the fibrous clay addition in the composite films was translated into an increase of the maximum degradation rate temperatures. For the Cat CNF films, the temperatures of maximum degradation rate were higher than those of the analogous films based on DES CNF and TEMPO CNF, similar to the trend described above for the onset temperature of degradation. In general, two peaks in the derivative curve were also observed. For the film with Cat CNF only, these peaks were at 314 and 345 °C, and for the composite films with 10 and 20% clay content, they were in the ranges of 302–313 and 341–349 °C, respectively. In some samples, these two peaks converged into only one peak at ca. 335 °C (films with 50% SEP B and 50% PAL). From these results, it therefore seems that the effect of the mineral on the thermal degradation rate of the films was minimal.

Char residue was ca. 25% for the neat DES CNF film, ca. 22% for the TEMPO CNF film, and ca. 18% for the Cat CNF film. As the mineral incorporation increases, the char residue also increases, with larger values obtained at higher clay content in the films (Table 4), as expected from the higher thermal stability of the mineral component. Accordingly, the highest char values were obtained for the films containing 50% clay (between 46 and 59%). Comparing films of the same mineral content, the DES CNF- and TEMPO CNF-based films always showed higher char values than the Cat CNF-based analogues. For instance, the char residues (at 1000 °C) for the films of DES CNF and TEMPO CNF were up to 36% and up to 43% with 10% mineral and 20% mineral, respectively; for the analogous films of Cat CNF, the char values only reached 26 and 33%, respectively. The addition of sepiolite, particularly for the DES CNF- and TEMPO CNF-based films, limits the release of volatile and combustible compounds during thermal degradation. This is a highly relevant result, considering the use of these composite films as flame-retardant materials.

### Costs of Films/Nanopapers Production

For implementation at an industrial level and commercialization of final products, the costs of production are a key factor. An assessment of the costs involved in the production of the films was made, and the results are summarized in Table 5. Chemicals (including raw materials) and energy costs were considered. In Table 5, film production costs are presented considering first only the values of the cost of material per square meter of film and then the additional energy costs involved in the preparation of the films during the vacuum filtration and drying steps for the total cost. The following considerations were taken into account for the estimation of the costs:

- The ChemSpider database was used to obtain the chemical prices. The lowest chemical prices among all the suppliers listed in this database were selected for the calculations.
- A clay price of 0.5 €/kg was considered.
- An energy cost of 0.1544 €/kWh and cellulosic pulp cost of 1.188 €/kg were assumed.
- The power consumption of the high-pressure homogenizer was 1.85 kW.
- The measured power consumption values for the vacuum pump and hot-press dryer were 0.16 kW and 0.42 kW, respectively.

**Table 5:** Costs Involved in the Production of CNFs (€/kg) and CNF Films (€/m^2^)

CNF	cellulosic pulp cost (€)	chemicals cost (€)^a^	high-pressure homogenization cost (€)	CNF cost (€/kg)	cost of film materials (€/m^2^)	total film cost (€/m^2^)
DES	1.19	143.11	9.52	153.81	6.15	6.74
DES + 10% clay					5.54	6.03^b^
DES + 20% clay					4.93	5.42^b^
DES + 50% clay					3.09	3.51^b^
TEMPO	1.19	27.26	9.52	37.97	1.52	1.94
TEMPO + 10% clay					1.37	1.78^b^
TEMPO + 20% clay					1.22	1.61^b^
TEMPO + 50% clay					0.77	1.12^b^
Cat	1.19	25.56	9.52	36.27	1.45	1.79
Cat + 10% clay					1.31	1.64^b^
Cat + 20% clay					1.16	1.49^b^
Cat + 50% clay					0.74	1.04^b^

a The detailed calculation of the chemicals cost can be found in Table S2.

b Calculations were made only for the films with SEP B.

The films prepared with DES CNF showed higher costs compared to those of TEMPO and Cat CNFs for the same mineral content in the film. The DES CNF exhibited higher production cost compared to TEMPO CNF and Cat CNF mainly because of the substantial amounts of sulfamic acid and urea required for the production of this CNF under the specific conditions applied (sulfamic acid/anhydroglucose molar ratio of 10:1 and sulfamic acid/urea molar ratio of 1:2). The cost of DES CNF could be reduced by reducing the excess of sulfamic acid used and by recycling the DES; however, these optimizations must be done without significantly compromising the transparency of the films. Due to the lower price of the minerals compared to the CNFs, the film cost was highly reduced by increasing the amount of mineral in the film. According to the estimations, the energy costs for the preparation of the neat DES CNF film and the corresponding composite films with SEP B are only around 10% of the material costs. For the analogous films with TEMPO CNF and Cat CNF, the ratio between the energy costs and the material costs is higher (>20%). In these films, the energy costs are lower compared to DES CNF films because of the lower filtration times (less energy) required; however, the ratio between energy costs and material costs for TEMPO CNF and Cat CNF films is higher due to the significantly lower material costs involved. It should be noted that in all cases the incorporation of clay mineral also reduces the filtration time, slightly reducing the energy cost associated with the filtration step. The energy costs associated with vacuum filtration could be eliminated by preparing the films using the solvent casting method, and the drying costs could also be significantly reduced through appropriate process optimization. The main factor limiting the composite film cost is therefore the CNF cost, particularly when working with chemically modified nanocelluloses. Other cheaper CNF types could be considered,^36^ but the transparency values would not be at the same levels as those achieved in the present study, namely, with DES and TEMPO CNFs. Notwithstanding, the fibrous clay incorporation clearly reduces the film’s price, and provided that the final properties are good, it is recommended. In this context, films with 50% mineral incorporation would be desirable, but at this clay content, only the DES CNF composite films with sepiolite showed the properties required for the purposes of the present work, i.e., films with high transparency, high strength, and good thermal stability.

## Conclusions

In this work, for the first time, composite films with high transparency were obtained from the admixture of very negatively charged and thin cellulose nanofibrils produced by a eutectic solvent pretreatment (DES CNF) and fibrous clay. Transparency values higher than 72% at the photopic wavelength were obtained for films prepared from DES CNF and 50% sepiolite content using a simple preparation method based on the dispersion of the components with a high-speed disperser. At 20% sepiolite content, transparencies of >83% were obtained. Acceptable results at 10–20% clay content were also obtained with a highly negatively charged TEMPO CNF, but not as good as those achieved with DES CNF. Additionally, of the two studied sepiolite samples (SEP A and SEP B), it was revealed that the sepiolite sample with easier dispersion in water (SEP B) also enabled films with better transparency, particularly in the series of the TEMPO CNF based-films. The remarkable transparency results achieved with the combination of DES CNF and sepiolite (slightly better with SEP B) can be attributed to the smaller particle size and better dispersion of DES CNF and SEP B in the mixture, allowing for the formation of denser and more compact films that scatter less radiation.

The mechanical properties of the composite films were not far from those of the neat CNF films. For the composite films of TEMPO CNF and sepiolite up to 20% clay content, the tensile strength was in the range of 58–77 MPa, close to the tensile strength of the neat CNF film (71 MPa). For the analogous films of DES CNF, the tensile strength decreased from 114 MPa to 58–88 MPa for 10–20% sepiolite, and for 50% sepiolite it was in the range of 63–89 MPa. The prepared composite films also showed good thermal stability, and the char residue was greatly increased by the mineral incorporation.

Overall, transparent and strong films could be obtained from DES CNF and sepiolite without the need for complex and expensive homogenization processes or chemical modifications of clay, which are commonly applied in the literature to produce transparent CNF–clay composite films. The combination of high strength, transparency, and good thermal properties of the described composite films and the cost reductions achieved by the use of simpler dispersion methods and the incorporation of inexpensive minerals render them highly suitable for several applications and facilitate their industrial implementation.

## Supplementary Material



## Notes

Procedures used for the characterization of DES CNF, TEMPO CNF, and Cat CNF; basis weight of the films of DES CNF, TEMPO CNF, and Cat CNF with sepiolite and palygorskite samples; cost of chemicals used for CNF production; transparency versus opacity of the obtained CNF–clay composite films; reflectance spectra of the CNF-SEP A and CNF-PAL composite films; Young’s modulus and elongation at break of the CNF–clay composite films; representative thermograms and derivative curves of films produced with DES CNF, TEMPO CNF, and Cat CNF (PDF)

## References

[1] Liu A., Walther A., Ikkala O., Belova L., Berglund L. A. (2011). Clay Nanopaper
with Tough Cellulose Nanofiber Matrix for Fire Retardancy and Gas
Barrier Functions. Biomacromolecules.

[2] Aulin C., Salazar-Alvarez G., Lindström T. (2012). High Strength, Flexible and Transparent
Nanofibrillated Cellulose–Nanoclay Biohybrid Films with Tunable
Oxygen and Water Vapor Permeability. Nanoscale.

[3] Wu C.-N., Saito T., Fujisawa S., Fukuzumi H., Isogai A. (2012). Ultrastrong
and High Gas-Barrier Nanocellulose/Clay-Layered Composites. Biomacromolecules.

[4] Wu C.-N., Yang Q., Takeuchi M., Saito T., Isogai A. (2014). Highly Tough
and Transparent Layered Composites of Nanocellulose and Synthetic
Silicate. Nanoscale.

[5] Alves L., Ferraz E., Gamelas J. A. F. (2019). Composites
of Nanofibrillated Cellulose
with Clay Minerals: A Review. Adv. Colloid Interface
Sci..

[6] Ruiz-Hitzky E., Darder M., Fernandes F. M., Wicklein B., Alcântara A. C.
S., Aranda P. (2013). Fibrous Clays
Based Bionanocomposites. Prog. Polym. Sci..

[7] Martín-Sampedro R., Eugenio M. E., Ibarra D., Ruiz-Hitzky E., Aranda P., Darder M. (2022). Tailoring
the Properties of Nanocellulose-Sepiolite
Hybrid Nanopapers by Varying the Nanocellulose Type and Clay Content. Cellulose.

[8] Alves L., Ramos A., Ferraz E., Ferreira P. J. T., Rasteiro M. G., Gamelas J. A. F. (2023). Design of Cellulose Nanofibre-Based
Composites with
High Barrier Properties. Cellulose.

[9] Gamelas J. A. F., Ferraz E. (2015). Composite Films Based
on Nanocellulose and Nanoclay
Minerals as High Strength Materials with Gas Barrier Capabilities:
Key Points and Challenges. BioResources.

[10] González
del Campo M. M., Darder M., Aranda P., Akkari M., Huttel Y., Mayoral A., Bettini J., Ruiz-Hitzky E. (2018). Functional
Hybrid Nanopaper by Assembling Nanofibers of Cellulose and Sepiolite. Adv. Funct. Mater..

[11] Ghanadpour M., Carosio F., Ruda M. C., Wågberg L. (2018). Tuning the
Nanoscale Properties of Phosphorylated Cellulose Nanofibril-Based
Thin Films To Achieve Highly Fire-Protecting Coatings for Flammable
Solid Materials. ACS Appl. Mater. Interfaces.

[12] Lisuzzo L., Wicklein B., Lo Dico G., Lazzara G., del Real G., Aranda P., Ruiz-Hitzky E. (2020). Functional
Biohybrid Materials Based
on Halloysite, Sepiolite and Cellulose Nanofibers for Health Applications. Dalton Trans..

[13] Ghanadpour M., Wicklein B., Carosio F., Wågberg L. (2018). All-Natural
and Highly Flame-Resistant Freeze-Cast Foams Based on Phosphorylated
Cellulose Nanofibrils. Nanoscale.

[14] Gupta P., Verma C., Maji P. K. (2019). Flame Retardant
and Thermally Insulating
Clay Based Aerogel Facilitated by Cellulose Nanofibers. J. Supercrit. Fluids.

[15] Tang J.-S., Lee C.-Y., Liao Y.-C. (2023). Chemical Resistant
Silver Nanowire/Cellulose
Nanofibril Flexible Transparent Conductive Coatings. Prog. Org. Coat..

[16] Zhu H., Xiao Z., Liu D., Li Y., Weadock N. J., Fang Z., Huang J., Hu L. (2013). Biodegradable
Transparent
Substrates for Flexible Organic-Light-Emitting Diodes. Energy Environ. Sci..

[17] Wu J., Che X., Hu H.-C., Xu H., Li B., Liu Y., Li J., Ni Y., Zhang X., Ouyang X. (2020). Organic Solar Cells
Based on Cellulose Nanopaper from Agroforestry Residues with an Efficiency
of over 16% and Effectively Wide-Angle Light Capturing. J. Mater. Chem. A.

[18] Du H., Parit M., Liu K., Zhang M., Jiang Z., Huang T.-S., Zhang X., Si C. (2021). Engineering Cellulose
Nanopaper with Water Resistant, Antibacterial, and Improved Barrier
Properties by Impregnation of Chitosan and the Followed Halogenation. Carbohydr. Polym..

[19] Henniges U., Angelova L., Schwoll S., Smith H., Brückle I. (2022). Microfibrillated
Cellulose Films for Mending Translucent Paper: An Assessment of Film
Preparation and Treatment Application Options. J. Inst. Conserv..

[20] Fukuzumi H., Saito T., Iwata T., Kumamoto Y., Isogai A. (2009). Transparent
and High Gas Barrier Films of Cellulose Nanofibers Prepared by TEMPO-Mediated
Oxidation. Biomacromolecules.

[21] Almeida R. O., Maloney T. C., Gamelas J. A. F. (2023). Production
of Functionalized Nanocelluloses
from Different Sources Using Deep Eutectic Solvents and Their Applications. Ind. Crops Prod..

[22] Almeida R. O., Ramos A., Håkonsen V., Maloney T. C., Gamelas J. A. F. (2024). Functionalized
Cellulose Nanofiber Films as Potential Substitutes for Japanese Paper. Carbohydr. Polym. Technol. Appl..

[23] Ming S., Chen G., He J., Kuang Y., Liu Y., Tao R., Ning H., Zhu P., Liu Y., Fang Z. (2017). Highly Transparent
and Self-Extinguishing Nanofibrillated Cellulose-Monolayer Clay Nanoplatelet
Hybrid Films. Langmuir.

[24] Liu Y., Yu S., Bergström L. (2018). Transparent
and Flexible Nacre-Like
Hybrid Films of Aminoclays and Carboxylated Cellulose Nanofibrils. Adv. Funct. Mater..

[25] Alves L., Ferraz E., Santarén J., Rasteiro M. G., Gamelas J. A. F. (2020). Improving
Colloidal Stability of Sepiolite Suspensions: Effect of the Mechanical
Disperser and Chemical Dispersant. Minerals.

[26] Ferraz E., Alves L., Sanguino P., Santarén J., Rasteiro M. G., Gamelas J. A. F. (2021). Stabilization
of Palygorskite Aqueous
Suspensions Using Bio-Based and Synthetic Polyelectrolytes. Polymers.

[27] Sirviö J. A., Ukkola J., Liimatainen H. (2019). Direct Sulfation of Cellulose Fibers
Using a Reactive Deep Eutectic Solvent to Produce Highly Charged Cellulose
Nanofibers. Cellulose.

[28] Saito T., Kimura S., Nishiyama Y., Isogai A. (2007). Cellulose Nanofibers
Prepared by TEMPO-Mediated Oxidation of Native Cellulose. Biomacromolecules.

[29] Lourenço A. F., Gamelas J. A. F., Nunes T., Amaral J., Mutjé P., Ferreira P. J. (2017). Influence of TEMPO-Oxidised Cellulose Nanofibrils on
the Properties of Filler-Containing Papers. Cellulose.

[30] Almeida R. O., Ramos A., Kimiaei E., Österberg M., Maloney T. C., Gamelas J. A. F. (2024). Improvement of the Properties of
Nanocellulose Suspensions and Films by the Presence of Residual Lignin. Cellulose.

[31] Alves L., Ramos A., Rasteiro M. G., Vitorino C., Ferraz E., Ferreira P. J. T., Puertas M. L., Gamelas J. A. F. (2022). Composite
Films
of Nanofibrillated Cellulose with Sepiolite: Effect of Preparation
Strategy. Coatings.

[32] Sehaqui H., Mushi N. E., Morimune S., Salajkova M., Nishino T., Berglund L. A. (2012). Cellulose Nanofiber
Orientation in
Nanopaper and Nanocomposites by Cold Drawing. ACS Appl. Mater. Interfaces.

[33] Honorato C., Kumar V., Liu J., Koivula H., Xu C., Toivakka M. (2015). Transparent Nanocellulose-Pigment Composite Films. J. Mater. Sci..

[34] González
del Campo M. M., Caja-Munoz B., Darder M., Aranda P., Vázquez L., Ruiz-Hitzky E. (2020). Ultrasound-Assisted Preparation of
Nanocomposites Based on Fibrous Clay Minerals and Nanocellulose from
Microcrystalline Cellulose. Appl. Clay Sci..

[35] Li P., Sirviö J. A., Hong S., Ämmälä A., Liimatainen H. (2019). Preparation of Flame-Retardant Lignin-Containing Wood
Nanofibers Using a High-Consistency Mechano-Chemical Pretreatment. Chem. Eng. J..

[36] Delgado-Aguilar M., González Tovar I., Tarrés Q., Alcalá M., Pèlach M. À., Mutjé P. (2015). Approaching
a Low-Cost Production of Cellulose Nanofibers for Papermaking Applications. BioResources.

