# Supplementary material for: High-Optical-Performance Composite Films of Deep Eutectic Solvent Pretreated-Cellulose Nanofibrils and Fibrous Clay Minerals
Source: Biomacromolecules. 2025 Sep 2;26(10):6430–43. doi: 10.1021/acs.biomac.5c00376 (PMC12818757; doi:10.1021/acs.biomac.5c00376)
Supplement: Supplementary file 1 [file bm5c00376_si_001.pdf]

# Supporting Information

## High-optical performance composite films of deep eutectic solvent pretreated-cellulose nanofibrils and fibrous clay minerals

*Ricardo O. Almeida[1], Eduardo Ferraz[2], Ana Ramos[3], Verner Håkonsen[4], Maria L. Puertas[5], José A. F. Gamelas[1*]*

[1]CERES, University of Coimbra, Department of Chemical Engineering, Rua Sílvio Lima, Pólo II, PT - 3030-790 Coimbra, Portugal

[2]TECHN&ART, Polytechnic Institute of Tomar, Quinta do Contador, Estrada da Serra, PT - 2300-313 Tomar, Portugal

[3]FibEnTech, University of Beira Interior, Department of Chemistry, Rua Marquês d'Ávila e Bolama, PT - 6201-001 Covilhã, Portugal

[4]NTNU NanoLab, Norwegian University of Science and Technology (NTNU), Trondheim 7491, Norway

[5]TOLSA, SA, Research & Technology for New Businesses, Ctra. de Madrid a Rivas Jarama, 35, ES - 28031 Madrid, Spain

**Characterization of the CNFs**

To determine consistency, approximately 10 g of each CNF suspension was weighed in duplicate, dried overnight in an oven at 105 °C, and the dried solid was then weighed. The consistency was calculated by dividing the mass of dried solid by the initial mass of the CNF suspension.

The degree of fibrillation was determined in duplicate by centrifuging 50 mL of CNF aqueous suspensions (0.05 wt%) at 9000 rpm for 30 min, using a Hettich Universal 32 centrifuge. After centrifugation, the supernatant was removed, and the retained fraction was dried at 105 ºC and weighed. The degree of fibrillation (%) was calculated by dividing the difference between the initial solid material and the retained solid by the mass of initial solid material.

The degree of polymerization (DP) of the CNF samples was estimated in duplicate from intrinsic viscosity (η) measurements performed in cupriethylenediamine solution at 25 ºC (ISO 5351:2010 standard). The DP was then calculated using the Mark-Houwink equation ($\eta = k \times DP^a$), with the following parameters: k = 0.42 and a = 1 (for DP < 950); k = 2.28 and a = 0.76 (for DP > 950)[1].

Depending on the type of chemical pretreatment applied, the content of the substituent group (and the corresponding degree of substitution) was determined by elemental analysis or conductimetric titration. The sulfate and cationic group contents of the sulfate CNF (DES CNF) and the cationic CNF (Cat CNF), respectively, were determined based on sulfur and nitrogen contents measured using an Elemental Analyser EA 1108 CHNS-O (Fisons). On the other hand, the carboxyl content of the TEMPO CNF was determined in duplicate by conductimetric titration. The TEMPO CNF suspension was acidified to a pH of ca. 3 using 0.01 M HCl and titrated with 0.01 M NaOH. The carboxyl content and the corresponding degree of substitution were then obtained from the conductimetric titration curves, as previously described in detail elsewhere[2].

The surface charge of the three CNF types was evaluated by zeta potential measurements on diluted CNF suspensions (0.05 wt%) using a Zetasizer Nano ZS (Malvern Instruments). Six measurements were performed for each CNF sample.

For the morphological characterization of the produced CNFs by atomic force microscopy (AFM), CNF films with a grammage of 20 g/m$^2$ were prepared by solvent

casting at room temperature. AFM imaging was performed on the top side of each film using a Bruker Dimension Icon AFM microscope at two different sites, with a scanning area of 1 × 1 $\mu m^2$, a resolution of 512 data points per line and a scan rate of 0.5Hz. The average nanofibril diameters for each CNF were measured from the captured AFM images using Gwyddion software, with 80 diameter measurements taken per film[3]

## References

(1) Henriksson, M.; Berglund, L. A.; Isaksson, P.; Lindström, T.; Nishino, T. Cellulose Nanopaper Structures of High Toughness. *Biomacromolecules* **2008**, *9*, 1579–1585. https://doi.org/10.1021/bm800038n.

(2) Lourenço, A. F.; Gamelas, J. A. F.; Nunes, T.; Amaral, J.; Mutjé, P.; Ferreira, P. J. Influence of TEMPO-Oxidised Cellulose Nanofibrils on the Properties of Filler-Containing Papers. *Cellulose* **2017**, *24*, 349–362. https://doi.org/10.1007/s10570-016-1121-9.

(3) Almeida, R.; Ramos, A.; Håkonsen, V.; Maloney, T.; Gamelas, J. Functionalized Cellulose Nanofiber Films as Potential Substitutes for Japanese Paper. *Carbohydr. Polym. Technol. Appl* **2024**, *8*, 100573. https://doi.org/10.1016/j.carpta.2024.100573.

## Results

**Table S1.** Basis weight of the films of DES CNF, TEMPO CNF, and Cat CNF with sepiolite and palygorskite samples.

| Film | Basis weight ($g/m^2$) |
|---|---|
| DES | 38.8 ± 0.1 |
| DES + 10% SEP A | 39.4 ± 0.3 |
| DES + 20% SEP A | 40.0 ± 0.0 |
| DES + 50% SEP A | 40.5 ± 0.3 |
| DES + 10% SEP B | 38.7 ± 0.7 |
| DES + 20% SEP B | 38.7 ± 0.8 |
| DES + 50% SEP B | 39.4 ± 0.1 |
| DES + 10% PAL | 38.1 ± 0.9 |
| DES + 20% PAL | 37.5 ± 1.4 |
| DES + 50% PAL | 37.8 ± 0.1 |
| TEMPO | 37.4 ± 0.8 |
| TEMPO + 10% SEP A | 38.1 ± 0.1 |
| TEMPO + 20% SEP A | 38.7 ± 0.4 |
| TEMPO + 50% SEP A | 38.4 ± 0.3 |
| TEMPO + 10% SEP B | 37.3 ± 0.5 |
| TEMPO + 20% SEP B | 37.5 ± 0.1 |
| TEMPO + 50% SEP B | 37.3 ± 1.0 |
| TEMPO + 10% PAL | 38.4 ± 0.3 |
| TEMPO + 20% PAL | 38.3 ± 0.1 |
| TEMPO + 50% PAL | 37.9 ± 0.4 |
| Cat CNF | 40.8 ± 0.1 |
| Cat + 10% SEP A | 38.4 ± 0.3 |
| Cat + 20% SEP A | 38.6 ± 0.3 |
| Cat + 50% SEP A | 39.1 ± 1.3 |
| Cat + 10% SEP B | 39.5 ± 0.7 |
| Cat + 20% SEP B | 40.6 ± 0.7 |
| Cat + 50% SEP B | 40.2 ± 0.1 |
| Cat + 10% PAL | 39.6 ± 0.6 |
| Cat + 20% PAL | 39.9 ± 0.6 |
| Cat + 50% PAL | 39.4 ± 1.3 |

*Measurements were made at 23 ºC and 50% RH

**Table S2.** Cost of chemicals used for CNF production.

| CNF | Chemicals | Amount of chemicals ($g_{che}/kg_{CNF}$) | Chemical prices (€/$kg_{che}$) | Cost of chemicals (€/$kg_{CNF}$) | Total cost of chemicals (€/$kg_{CNF}$) |
|---|---|---|---|---|---|
| DES | Sulfamic acid | 5990 | 13.92 | 83.38 | **143.11** |
| | Urea | 7410 | 8.06 | 59.72 | |
| TEMPO | TEMPO | 16 | 110.86 | 1.77 | **27.26** |
| | NaBr | 100 | 32.07 | 3.21 | |
| | NaClO | 6710 | 3.32 | 22.28 | |
| Cat | CHPTAC | 580 | 38.61 | 22.39 | **25.56** |
| | NaOH | 493 | 6.43 | 3.17 | |

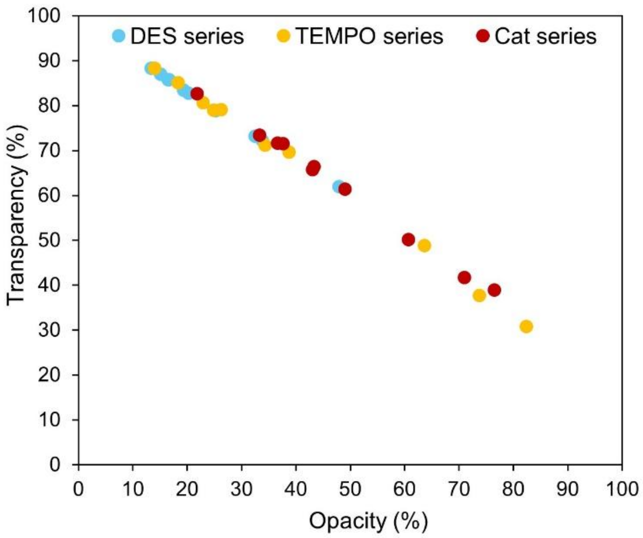


**Figure S1** – Transparency vs. opacity of the obtained CNF-clay composite films.

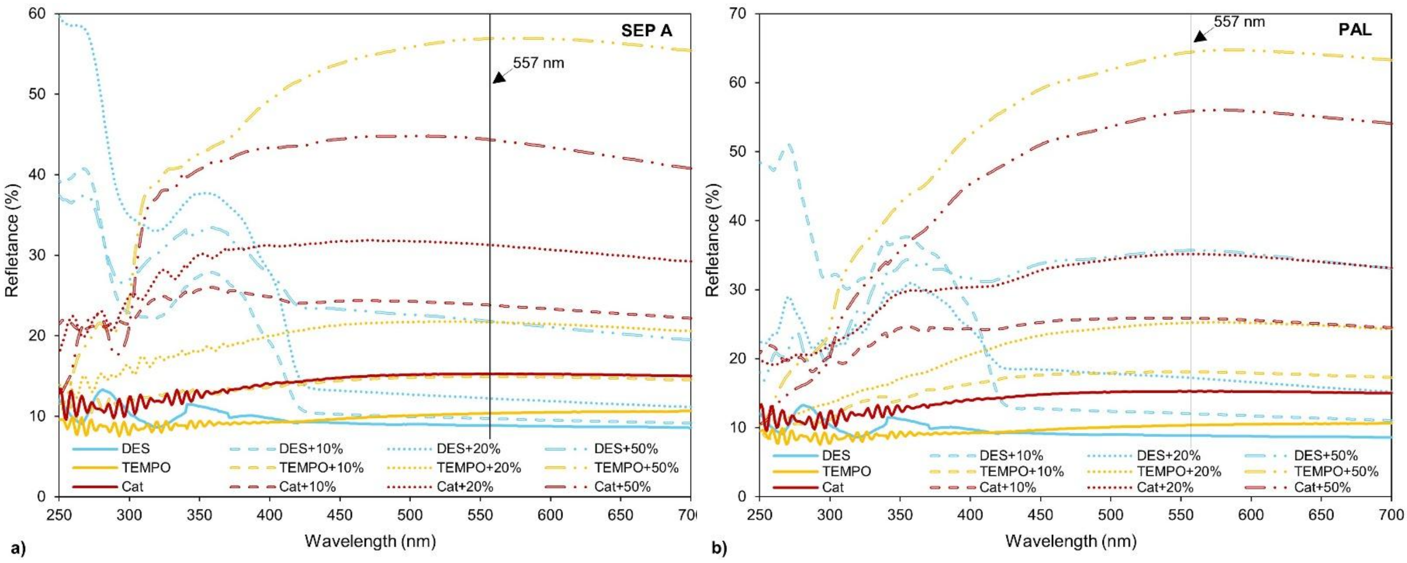


**Figure S2** – a) Reflectance spectra of the CNF-SEP A composite films; b) Reflectance spectra of the CNF-PAL composite films.

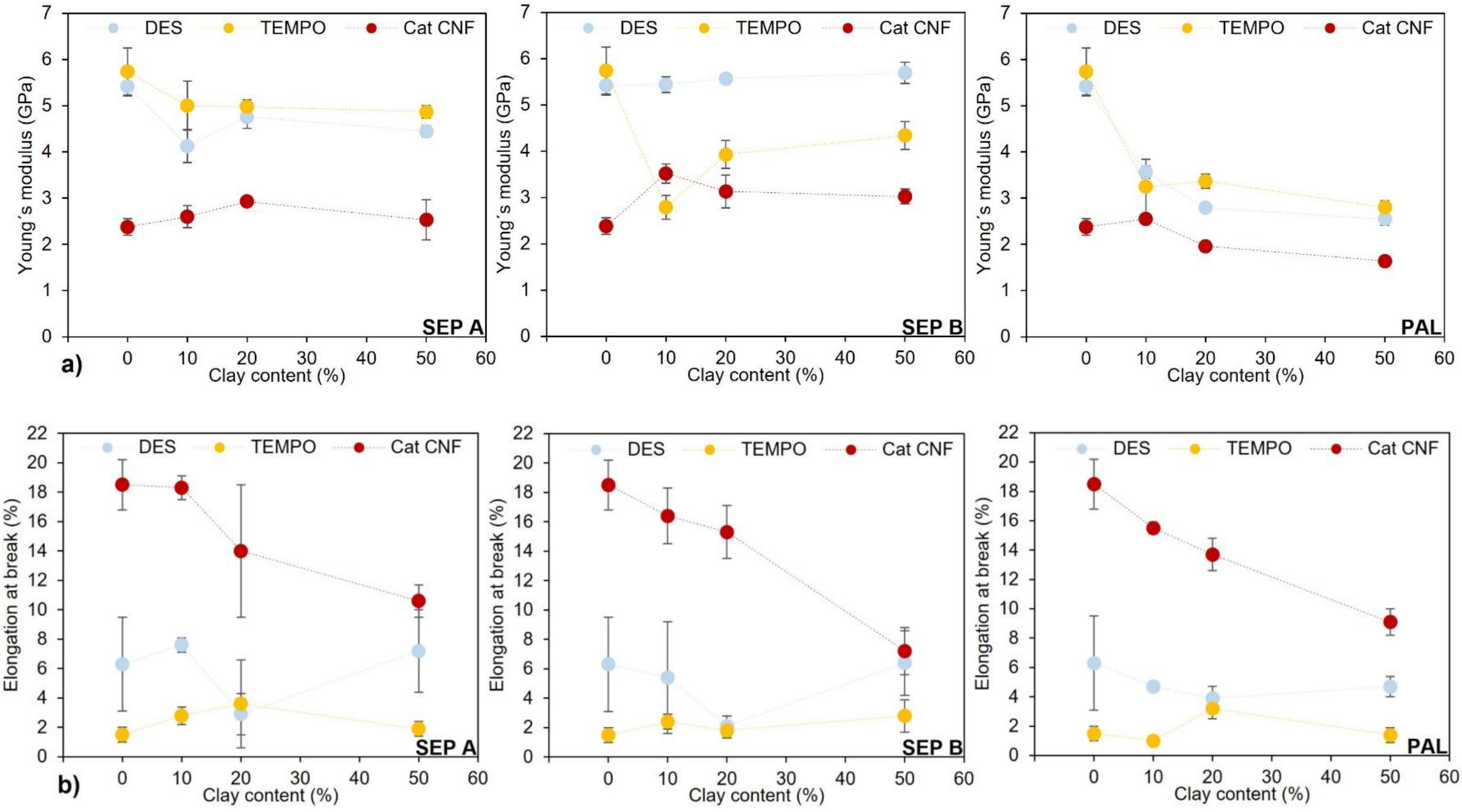


**Figure S3**. Young’s modulus (a) and elongation at break (b) of the CNF-clay composite films (23 ºC and 50% RH).

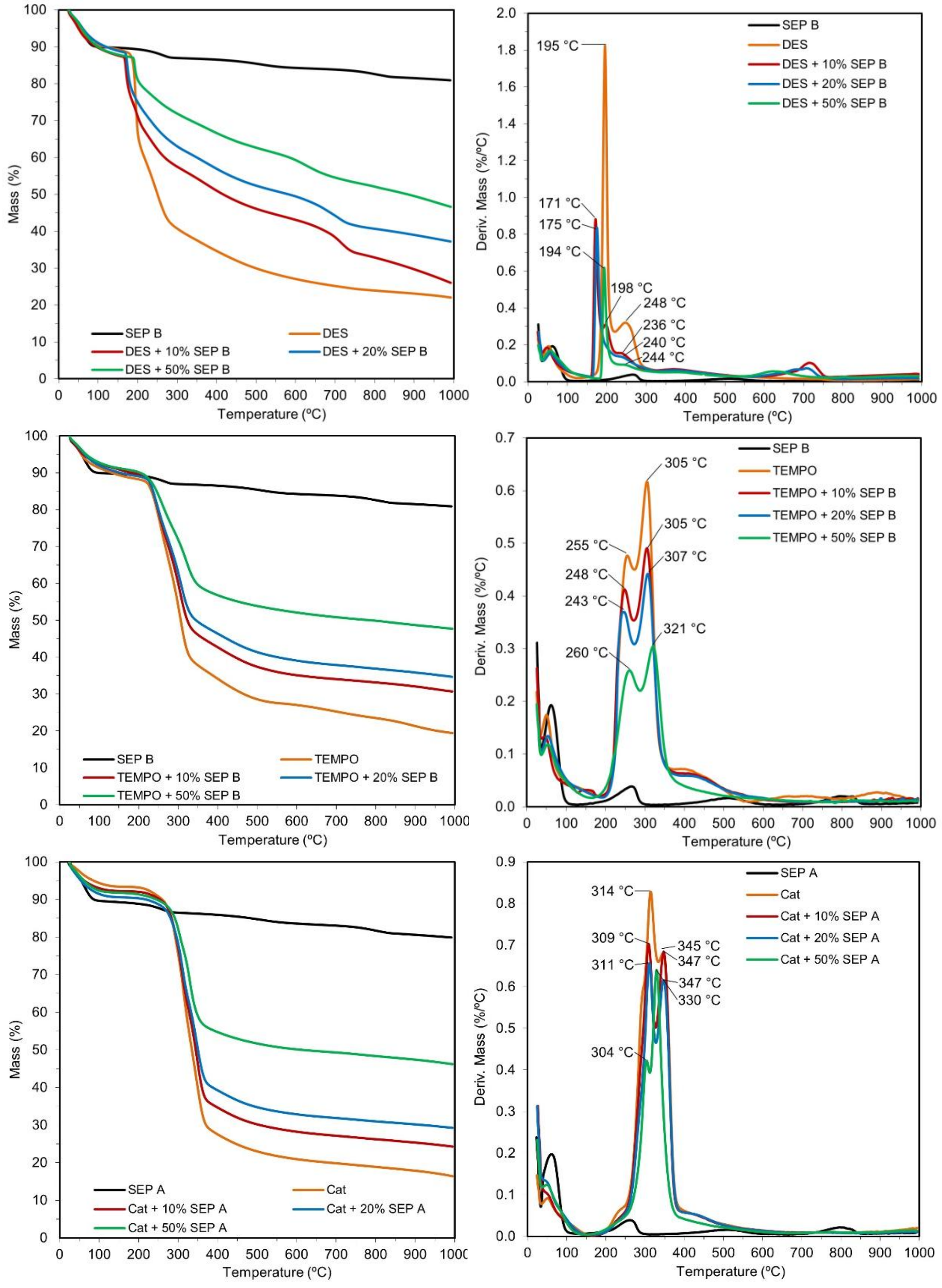


**Figure S4** – Representative thermograms and derivative curves of films produced with DES CNF, TEMPO CNF, and Cat CNF.
